# Regulation of platelet-activating factor-mediated protein tyrosine phosphatase 1B activation by a Janus kinase 2/calpain pathway

**DOI:** 10.1371/journal.pone.0180336

**Published:** 2017-07-07

**Authors:** Geneviève Hamel-Côté, Daniel Gendron, Marek Rola-Pleszczynski, Jana Stankova

**Affiliations:** Immunology Division, Department of Pediatrics, Faculty of Medicine and Health Sciences, Université de Sherbrooke, Sherbrooke, QC, Canada; Wayne State University, UNITED STATES

## Abstract

Atherosclerosis is a pro-inflammatory condition underlying many cardiovascular diseases. Platelet-activating factor (PAF) and interleukin 6 (IL-6) are actively involved in the onset and progression of atherosclerotic plaques. The involvement of monocyte-derived macrophages is well characterized in the installation of inflammatory conditions in the plaque, but less is known about the contribution of monocyte-derived dendritic cells (Mo-DCs). In the same way, the involvement of calcium, phospholipase C and A2 in PAF-induced IL-6 production, in different cells types, has been shown; however, the importance of the Jak/STAT pathway and its regulation by protein-tyrosine phosphatases in this response have not been addressed. In this study, we report that PAF stimulates PTP1B activity via Jak2, thereby modulating PAF-induced IL-6 production. Using HEK 293 cells stably transfected with the PAF receptor in order to discriminate the pathway components, our results suggest that Jak2 modulates PAF-induced IL-6 production via both positive and negative pathways. Jak2 kinase activity was necessary for maximal transactivation of the IL-6 promoter, as seen by luciferase assays, whereas the same kinase also downregulated this promoter transactivation through the activation of a calcium/calpain/PTP1B pathway. The same pathways were operational in monocyte-derived dendritic cells, since PAF-induced PTP1B activation negatively regulated PAF-induced IL-6 mRNA production and, in addition, Jak2 activated calpain, one of the components involved in PAF-induced PTP1B activation. Results obtained in this study indicate that Jak2 activation is important for maximal IL-6 promoter transactivation by PAF and that PTP1B is involved in the negative regulation of this transactivation. However, PTP1B does not directly regulate Jak2 activation, but rather Jak2 regulates PAF-induced PTP1B activation.

## Introduction

Platelet-activating factor (PAF) is a potent lipid mediator involved in different physiological processes, ranging from working memory [1], to pain [2] and inflammation [3]. Consequently, PAF is involved in many pathophysiological states such as ovarian cancer [4] and atherosclerosis [5]. PAF is found in atherosclerotic plaques [6] and in arteries of patients with severe atherosclerosis [7]. Interestingly, this lipid mediator is involved in both the onset and progression of atherosclerosis, in terms of cell migration, adhesion, in addition to cytokine and chemokine production [5]. It is also involved in induction of metalloproteases which are known to destabilize the plaque [8]. Among PAF-induced cytokines, IL-6 contributes to atherosclerosis, since a moderate but sustained elevation of serum IL-6 levels correlates with an increased risk in coronary heart disease [9]. In addition, at a cellular level, IL-6 stimulates endothelial cells, facilitating leukocyte recruitment to coronary lesions via an increase in chemokine production and surface expression of ICAM-1 [10]. Moreover, inhibition of IL-6 trans-signaling seems to decrease lipid deposition and lesion size in *Ldlr* ^-/-^ mice on high-fat and high-cholesterol diet, leading to regression of advanced atherosclerosis [11]. IL-6 inhibition also prevents macrophage infiltration and increase of adhesion molecules expressed by endothelial cells [11]. IL-6 production by macrophages found in atherosclerotic plaques is well characterized [6], but less is known about its production by immature DCs. Given that immature DCs are among the first cells found in arterial localizations susceptible to plaque development [12], that they respond to PAF, one of the first mediators produced by activated endothelium [5], and that PAF can induce IL-6 production [13], it is important to investigate the molecular mechanisms that regulate IL-6 production by DCs during the early stages of plaque formation.

Signalling pathways leading to PAF-induced IL-6 production have been well characterized in different cells. PAFR, a G-protein coupled receptor (GPCR) is able to transduce PAF-induced signals via both Gα_i_- or Gα_q_-protein-dependent pathways [14–15] in addition to G-protein-independent pathways [16–15]. Among PAF-induced signalling pathways leading to IL-6 production, studies point to the involvement of phospholipase C (PLC) and cytosolic phospholipase A2 (PLA_2_) [17], 5'-lipoxygenase [18] and store-operated channel (SOC) [19] or voltage-gated L-type channel-dependent [17] calcium pathways. PAF is also known to initiate pathways leading to activation of NFκB [20–21] and CCAAT/enhancer binding proteinβ (C/EBPβ) [22], transcription factors known to bind and activate the IL-6 promoter [23–24]. Among other transcription factors involved in IL-6 production, STAT1 has been demonstrated to modulate IL-6 production either by induction [25–26] or interaction [27] with IRF-1 which can directly bind to the hIL-6 promoter [28]. In addition, STAT5 seems to also be able to modulate IL-6 production, given that the LPS-induced binding of STAT5 to the IL-6 promoter, in association with NFκB p50, leads to increased promoter activation in comparison to the absence of STAT5 [29]. The NFκB binding capacity to the NFκB element is also increased in the presence of overexpressed STAT5A in response to erythropoietin and the authors of this report also demonstrated that the constitutive activation of STAT5A is sufficient to trans-activate the IL-6 promoter if the NFκB sites are present [30]. We have shown that PAF can activate Jak2 [31] and Tyk2 leading to STAT1, STAT2 and STAT5 tyrosine phosphorylation [16], whereas STAT3 can be phosphorylated by Tyk2- and Src-dependent pathways.

We were interested in studying the involvement of the Jak/STAT pathway in PAF-induced IL-6 production and the signaling mechanisms that could regulate it. Negative regulation of the Jak/STAT pathway is important for resolving cytokine-induced inflammation and could be instrumental in stopping progression of atherosclerosis. Many pathways could take part in negative regulation of the Jak/STAT pathway in an atherosclerotic context. In particular, protein tyrosine phosphatases (PTPs) regulating the Jak/STAT pathway are also associated with atherosclerosis since certain SNPs in *PTPN1*, the coding gene for PTP1B, correlate with protective effects on coronary arterial disease in a Han Chinese population [32], whereas others are associated with an increase in calcification of coronary arteries in Caucasians [33]. At a cellular level, PTPs can regulate the specificity of signal transduction, since after stimulation with gamma interferon, a pro-inflammatory cytokine known for its contribution to atherogenesis, the phosphorylation levels of Jak1 are increased in SHP-2-knockout cells [34], whereas Jak2 phosphorylation levels are increased in MEF knockouts for PTP1B [35].

Among signaling molecules that could regulate PAF-induced Jak/STAT pathway, PTPs seem to be good candidates, since previous results have suggested their involvement in regulation of PAF-induced PGE_2_ production, endocytosis and cell-surface expression of PAFR [36–37]. Among PTPs, PTP1B could a potential regulator of PAF-induced IL-6 production, since it can directly bind, dephosphorylate and inactivate both Jak2 and Tyk2 [35]. PTP1B is an endoplasmic reticulum-bound PTP mostly known for its modulation of tyrosine kinase receptor signaling of insulin [38], leptin [39], PDGF [40] or Colony-Stimulating Factor [41] receptors. In addition, PTP1B has been shown to be involved in the regulation of TLR-induced IL-6 production in the mouse macrophage cell line, Raw 267 [42].

Altogether, these data led us to investigate the involvement of PTP1B in PAF-induced IL-6 production.

## Materials and methods

### Reagents and chemical products

PAF (C-16), calpeptin and rabbit anti-N-Terminal PAFR were from Cayman Chemicals (Ann Arbor, MI, USA). Thapsigargin, PTP1B inhibitor, AG490, Jak2 inhibitor IV and Jak2 inihitor III (SD-1029) were from Calbiochem (San Diego, CA, USA). BSA for cell-culture, cOmplete, Mini Protease Inhibitor Tablets and ATP for luciferase assays were from Roche Diagnostics (Laval, QC, Canada). Dulbecco’s Modified Eagle Medium (DMEM, high glucose), GST-FER, 4′,6-diamidino-2-phenylindol dilactate (DAPI), Fluoro3 and D-Luciferin Na+ Salt were from Invitrogen (Burlington, ON, Canada). Fetal bovine serum (FBS) was from PAA Laboratories (Etobicoke, ON, Canada). TransIT-LT1 transfection reagent was from Mirius Bio (Medicorp Montréal, QC, Canada). Antibodies: Rabbit anti-Jak2 for microscopy (clone C20), rabbit anti-PTP1B (clone H135) (eptiope 301–435), mouse anti-PTP1B (H135) (eptiope 301–435) were from Santa-Cruz (LaJolla, CA, USA). Goat anti-PTP1B (epitope 2–321) was from R&D Systems, Minneapolis, MN. Anti-Src and pTyr 527 Src were from Cell Signalling (New England Biolabs Ltd., Pickering, ON, Canada). Rabbit anti-Jak2 and rabbit anti-phospho-Jak2 were from EMD Millipore (Mississauga, ON, Canada). Mouse anti-CD83 and mouse anti-CD86 were from BD Pharmingen, Mississauga, ON, Canada. Calpain Fluorometric Activity Assay Kit was from Biovision (Milpitas, CA, USA). Poly(Glu-:Tyr) [4:1], Gly-Gly, anti-FLAG and BSA essentially IgG-free were from Sigma-Aldrich, (Oakville, ON, Canada). Amersham ECL Select Western Blotting Detection Reagent, G-25 Sephadex PD-10 and GSH-sepharose beads, nitrocellulose membranes (Hybond ECL) were from GE (GE Heathcare, Piscataway, NJ, USA). Vectashield Mounting Media was from Vector Laboratories (Burlington, ON, Canada). Guanidine was from Fisher Scientific, (Nepean, ON, Canada) and p-Nitrophenyl phosphate (pNPP) was from ProteoChem (Loves Park, IL, USA). RPMI 1640 was from Wisent (Saint-Bruno, QC, Canada). RhGM-CSF and rhIL-4 were from Peprotech (Rocky Hill, NJ, USA)

### Plasmids

Clones from E-coli populations with human PTP1B received from Open Biosystems (Ge Dharmacon, Lafayette, CO, USA) were isolated and DNA was extracted. PCR products were inserted in the pcDNA3 poly-cloning site after KpnI/NheI vector digestion. PTP1B sequence was also cloned in pCDNA3-FLAG, pGFP2-N3(h)_linker (a gift of Dr. C. Le Gouill, U. Montréal, Montreal, Canada) or pCDNA3-Venus. Integrity of the construct was confirmed by sequencing (Centre d'innovation Génome Québec and McGill University, Montreal, Canada). Jak2 and kinase-dead mutant K882E Jak2 were a gift from Dr Roy J. Duhé (University of Mississippi, USA). pGL3-IL-6 plasmid was described elsewhere [23].

### siRNAs sequences

We used a mix of 3 siRNA duplexes that we designed using siRNA Wizard Guideline (http://www.sirnawizard.com) and Ambion website. First one is against the mRNA region 338–357 (5'-AUAGGUACAGAGACGUCAGUU-3'), the second, against region 702–720 (5'-CCAAGAAACUCG AGAGAUC-3') and the last one, against the region 2038–2056 (5'-AUCCUCAGGUAGUACUGGGU U-3'). SiRNAs duplexes, with a UU overhang and siRNAs controls (mission siRNA Universal Negative control #1) were from Sigma-Aldrich (Oakville, ON, Canada).

### Cell culture

HEK-293 (CRL. 1573, American Type Culture Collection (ATCC), Rockville, USA) stably transfected with pIRES_puro_PAFR_HA (HEK-PAFR) were grown in DMEM, 5% FBS at 37°C 5%C0_2_ in a humidified atmosphere with puromycin, 5μg/ml, penicillin, 60 μg/ml and streptomycin, 100 μg/ml.

Monocyte-derived dendritic cells (Mo-DCs) were generated from monocytes obtained from healthy volunteers after informed, written consent in accordance with protocol #93–04 approved by the Comité d'Éthique de la recherche chez l'Humain (Human ethics research committee), Université de Sherbrooke. Monocytes were isolated from venous peripheral blood after enrichment by dextran sedimentation followed by purification by density gradient centrifugation on Ficoll-Hypaque and after adhesion on autologous serum-coated Petri dishes. Following intensive washings, adherent cells were recovered and cultured for 7 days in RPMI 1640 supplemented with 10%FBS, rhGM-CSF (20 ng/mL) and rhIL-4 (20 ng/mL) at 1.6x10^6^ cells/ml in a 6-well plate, 3 ml/well. Medium was changed after 24–48h by removing half of the volume and adding the same volume of new medium. At day 5, the medium was changed by removing 1.5mL, but refilling with medium containing only 10ng/mL of each cytokine. Finally, at the end of day 6, 3ml of RPMI 5% FBS without any cytokine was added to begin the starvation of the cells. On day 7, cells were collected and stimulated as described below. Where mentioned, cells were transfected with siRNAs: at day 4. 75μl of a 2μM siRNA solution (in RPMI 1640) containing 2.3μL TransiT LT1 was added to 4.8x10^6^ cells (1 well of a 6 wells-plate, 1.5mL) and incubated at 37C, 5% CO_2_. Thereby, the siRNA final concentration is 100nM. After 6h, 1.5mL of fresh medium with 20ng/ml of each cytokine and 10%FBS was added. A second transfection of siRNAs was done 30h after the first one: 75μl of a solution of 1.53μM siRNA in RPMI 1640 containing 2.3μL TransiT LT was added to 4.8x10^6^ cells (1 well of a 6-wells plate, 1.5mL) and incubated at 37C, 5% CO_2_. Thereby, the siRNA final concentration is 76nM. After 6h, 1.5mL of fresh medium with 10ng/ml of each cytokine and 10%FBS was added.

### Transient transfection and luciferase assays

HEK-PAFR were plated 12 hours before transfection in 24-wells plates. Cells were transiently transfected with 200ng of pcDNA3-hPTP1B and 40ng of pGL3-hIL-6 per well using 0,75μl of TransIT LT1 transfection reagent according to the manufacturer’s instructions. After 8h in DMEM 5% FBS without puromycin, medium was changed and cells were incubated in DMEM 0.2% BSA for 16h. The cells were lysed 6h after stimulation by PAF at indicated concentrations. Where mentioned, cells were pre-treated for 20min with the inhibitors at indicated concentrations. Luciferase activity in lysates was measured as described before [43] using a Sirius luminometer (Berthold detector Systems, Montreal, QC, Canada).

### Western blot analysis

HEK-PAFR were plated 12 hours before transfection in 6-well plates. Where mentioned, cells were transiently transfected with 625ng of pcDNA3-human WT or mutant D181A PTP1B per well using 3μl of TransIT LT1 transfection reagent according to the manufacturer’s instructions. After 8h in DMEM 5% FBS without puromycin, medium was changed and cells were left in DMEM 0.2% BSA for 16h. Cells were then stimulated with 100nM PAF for indicated times and the reaction was stopped on ice with ice-cold PBS containing 4μM Na_3_VO_4_ and 20mM NaF. Cells were scraped, centrifuged and lysed by adding ice-cold lysis buffer containing inhibitors (50 mM Tris HCl, pH 7.4, 150 mM NaCl, 1 mM EDTA, 1% TRITON X-100, 2mM NaF, 4mM Na_3_VO_4_, 10nM calyculin A and cOmplete, Mini Protease Inhibitor Tablet) for 20 min on ice before being frozen at -80°C.

When the samples were thawed, they were centrifuged, 4x loading buffer (40% glycerol, 258mM Tris-HCl pH 6.8, 8% SDS, 0,008% bromophenol blue, 20% 2-mercaptoetanol) was added to the supernatants, proteins were separated on 10% SDS-PAGE and subsequently transferred to a nitrocellulose membrane (Hybond, GE). Membranes were blocked with 5% milk in Tris-buffered saline (TBS) with 0.1%Tween 20 (TBS-T) before overnight blotting at 4°C in TBS-T 5% milk with appropriate antibodies. For phospho-proteins, membranes were washed 3x5min in TBS-T after blocking in milk, and blocked again for 20min in TBS-T 5% BSA before overnight blotting at 4°C in BSA. Finally, HRP-coupled secondary antibody (Cell Signalling) was added for 1h, protein expression was revealed by ECL chemiluminescence detected with Versadoc (BioRad). Signal intensity was analyzed with ImageJ 1.43X software (National Institutes of Health, Bethesda, MD, USA).

### Microscopy

HEK-PAFR were grown overnight on poly-L-lysine coated coverslips in DMEM, 5%FBS, transfected with 400ng Venus-tagged pCDNA3-D181A-hPTPN1 and 1.5μL Transit LT1. After 24h, cells were starved 7h in DMEM 0.2% BSA and stimulated with 100nM PAF for indicated times. Cells were fixed with 2.5% paraformaldehyde (PFA) for 15min at room temperature (RT), then placed in 0.5% (v/v) Triton X-100 in PBS for 10min RT, blocked 20min in PBS 3%BSA, 0.1%Triton X-100 and incubated overnight, 4°C, in PBS, 1%BSA, 0.1% Triton X-100 containing anti-Jak2 antibodies (Abs) (C20, Santa-Cruz, 1:200). Controls with rabbit isotype Abs were performed in parallel. The coverslips were washed 3 times and incubated 1h, RT, in PBS, 2% BSA, 0.1% Triton X-100 containing anti-rabbit-Cy5 Abs (1:500, Jackson Immunoresearch). After washing, the coverslips were mounted on slides using Vectashield containing DAPI. Acquisitions were performed with an Plan Apo 60x oil immersion objective NA 1.42 on inverted spectral scanning confocal microscope FV1000 (Olympus, Tokyo, Japan). Images were acquired the same day, typically from 4 different optical fields for each experimental condition using identical settings of the instrument. Images were analyzed with FluoView software (Olympus). The percentage of colocalized pixels (P) was calculated

$$\%Pixels=\left(1-\frac{\left(PPTP1Btotal-PColocalizedPTP1B\right)}{PColocalizedPTP1B}\right)\times 100$$

### In-gel PTP assays

In-gel PTP assays were performed as described elsewhere [44–45]. Briefly, 2x10^6^ cells were starved for 3h in serum-free medium with 0.2%BSA and stimulated for indicated times. Stimulation was stopped with PBS containing NaF (final concentration 20mM) and Na_4_VO_3_ (final concentration 5mM). Cells were collected and lysed 20min on ice with 80μL in-gel PTP lysis buffer (0.5% NP-40, 50mM HEPES (pH 7.4), 150mM NaCl, 1mM EDTA, 2mM EGTA, 1mM Na_4_VO_3_ 1mM NaF, cOmplete, Mini Protease Inhibitor Tablet) and then centrifuged. 27μL of 4x loading buffer was added to cleared lysate. Lysates were frozen until the assay was performed. For immunoprecipitation, cleared lysates were incubated 30min at 4°C with Protein-A sepharose beads and centrifuged. Supernatants were incubated 3h with rabbit anti-PTP1B Abs (1.4μg) or rabbit isotype control Abs before addition of 25μL of a solution 50% protein-A sepharose beads. Lysates were then incubated for 2h at 4°C. Beads were recovered by centrifugation (3000rpm, one minute) and subsequently rinsed three times with ice-cold TBS-T. Beads were resuspended in 2x loading loading buffer.

Poly(Glu-:Tyr) [4:1] (1.2mg) was labelled overnight at RT using GSH-Sepharose beads- coupled GST-FER and 0.5mCi [y-^32^P] ATP (3000 Ci/mmol) in incubation buffer (50mM immidazole, pH 7.2, 30mM MgCl_2_, 10mM MnCl_2_, 10mM DTT, 1mM Na_3_VO_4_, 1% Triton and 0.1% 2-mercaptoethanol). Poly(Glu-:Tyr) [4:1] was then purified on G-25 Sephadex PD-10 by collecting 1mL fractions eluted with a 50mM immidazol buffer (pH 7.2) and quantification was performed using a LKB-Wallac β-counter. Fraction with the highest activity was aliquoted and stored at -20°C.

In-gel PTP assays were performed by copolymerizing the labelled poly(Glu-:Tyr) at a ratio of 1.5x10^6^cpm for 20mL in a 10% acrylamide SDS-PAGE gel, overnight. 25μg of proteins from HEK-PAFR lysates and 15μg from Mo-DCs lysate were used and an equivalent amount of immunoprecipited PTP1B was used. After migration, gels were fixed (50mM Tris-HCl, pH 8.0, 20% isopropanol) overnight, washed 2x30min in washing buffer (50m Tris-HCl pH 8.0, 0,3% 2-mercaptoéthanol) and left for 90min in renaturing buffer (50mM Tris-HCl, pH8.0, 0,3% 2-mercaptoethanol 6M guanidine). After that, gels were washed 2x1h in assay buffer (50mM Tris-HCl, pH 8.0, 0,04% (v/v) Tween 40, 1mM EDTA, 0.3% 2-mercaptoethanol), incubated 1h in assay buffer with 3mM DTT and changed once again for the assay buffer with 3mM DTT. Gels were incubated overnight at RT, stained with Coomassie Blue and dried under vacuum. Dried gels were exposed to Hyperfilm MP (Amersham Pharmacia Biotech). Signal intensity and protein loading obtained with Coomassie blue staining was quantified by densitometry with Epson Expression 1680 Scanner and analyzed with ImageJ 1.43X software, NIH. Data are presented as normalized density. This value is calculated in two steps. First a value is calculated with the ratio of the density for the band X with a molecular weight Y over the density obtained with the Coomassie blue staining for the entire lane. Similarly, a value is calculated for the band with the same molecular weight found in unstimulated cells. Secondly, the normalized density is calculated with the ratio of the calculated value for the band X, found in step one, over the calculated value obtained for the corresponding band in unstimulated cells.

### Colorimetric PTP assays

Colorimetric PTP assays were performed with a protocol adapted from T. McAvoy and C. Nairn [46]. Pre-treated and stimulated HEK-PAFR were lysed 20min on ice with lysis buffer (50 mM Tris HCl, pH 7.4, 150 mM NaCl, 1 mM EDTA, 1% Triton X-100, 10mM NaF, 1 mM Na_3_VO_4_, 10μM Calyculin A and cOmplete, Mini Protease Inhibitor Tablet) and frozen at -80°C. PTP1B was immunoprecipitated from cleared lysates (at least 1mg of lysate) as described for in-gel PTP assays. Instead of being resuspended in loading buffer, beads were resuspended in 130μL PTP buffer (25mM HEPES, pH7.4, 0.1mM EDTA, 100μg/mL BSA, 0.01% Tween 20, 1mM DTT), distributed in 96-wells plate (30μL/wells) and incubated 10min at RT. After that, 20μL of substrate is added (pNPP, 95mg/mL in PTP buffer). Plates are incubated at 30°C under mild agitation. Optical density (OD) is taken every 10min at 405nm (reference 800nm) until the signal is saturated. OD is plotted as function of time and dephosphorylation rates are taken in linear portion of the curve. Hydrolysis rate is calculated with an extinction coefficient of 18000M^-1^cm^-1^.

### Calpain assays

Calpain activity was determined with Fluorometric Activity Assay Kit according to the manufacturer's instructions. Briefly, 1 plate with 6-wells of HEK-PAFR or 2x10^6^ DCs was pre-treated and stimulated as mentioned above, then lysed in 100μL of lysis buffer 1min before centrifugation. Protein concentration was determined by the Bradford assay (Pierce). 80–100μg of DCs lysate or 200μg of HEK-PAFR lysate was used for determination of calpain activity. Calpain activity levels were determined by fluorescence (Excitation wavelength: 355nm, Emission wavelength: 405nm) read with a PerkinElmer Fusion spectrometer.

### Calcium assays

HEK-PAFR were plated in 96-well plates and grown until confluence. Cells were incubated 30min with 5μM Fluo3 calcium probe and washed 3x with HBSS 0,5% BSA without calcium. Cells were then incubated for 20min in HBSS 0.2%BSA with Jak2 inhibitor IV or SD-1029. CaCl_2_ (2mM final) or buffer without CaCl_2_ was added to cells, after reading of the fluorescence basal levels, the cells were stimulated as indicated. Minimal and maximal fluorescence was determined by addition of EGTA (100mM) or Triton X-1000 (0.5% final), respectively. Data were obtained by acquiring fluorescence with a 140ms integration time with 20s between each reading taken on an Ascent Fluoroskan (Thermo Scientific).

### Flow cytometry analysis

Mo-DCs were collected, fixed with 2% PFA for 15min RT and non-specific binding sites were blocked by incubating cells for 30min in PBS 2%BSA containing human IgG (5μg/mL). Cells were stained with mouse anti-CD83 Abs (1:200) or with mouse anti-CD86 Abs (1:200), 1h at RT or overnight with rabbit anti-N-terminal PAFR Abs (1:250) in PBS 2% BSA, IgG 1μg/mL. After washes with PBS, cells were stained with secondary antibody coupled to Cy5 for 45min. After washing, cells were analyzed on a FACSCalibur flow cytometer using the CellQuestPro software.

### RNA isolation and real-time quantitative PCR

RNA was obtained using Trizol reagent (Invitrogen, Burlington, ON, Canada) according to the manufacturer’s instructions. After quantification, 1.0 μg of RNA was converted to cDNA, QuantiTect Reverse Transcription Kit, according to the manufacturer's instruction. GAPDH, IL-6 and PTP1B expression was measured using real-time PCR performed on a Rotor-Gene 3000 (Corbett Research, Kirkland, QC, Canada) as described previously [13]. The following oligonucleotide primer sets were obtained from IDT (Coralville, Ind, USA): human GAPDH: forward, 5'-GATGACATCAAGAAGGTG GTGAA-3' /reverse, 5'-GTCTTACTCCTTGGAGGCCATGT-3'; human IL-6: forward, 5'-GTGTGA AAGCAGCAAAGAGGC-3'/reverse, 5'-CTGGAGTACTCTAGGTATAC; human PTP1B: forward, 5'-acagagtgatggagaaaggttc-3'/reverse, 5'-ctcgAgtttcttgggttgtaag-3'. Gene expression was normalized with GAPDH mRNA content and differences were calculated with the delta-delta (ΔΔ)Ct method according to the following formula: (ΔΔCt = [(Ct G.O.I.Ctl −Ct HK.G.Ctl) − (Ct G.O.I.STIM. −Ct HK.G.STIM.)]. Comparison of the expression of each gene between its control and stimulated/siRNA transfected states was determined by ΔΔCt. Results were then transformed into fold variation measurements: fold increase -2ΔΔCt.

### Statistical analysis

Statistical significance was calculated using Prism 6 software (GraphPad Software, San Diego Calif, USA). For analysis of differences between experimental groups, we used Student's t-test, paired one-way and paired two-way ANOVA with appropriate post-test, as described in figure legends. Values of *P* ≤ 0.05 were considered statistically significant.

## Results

### PAF regulates PTP1B activity

**Fig 1:**
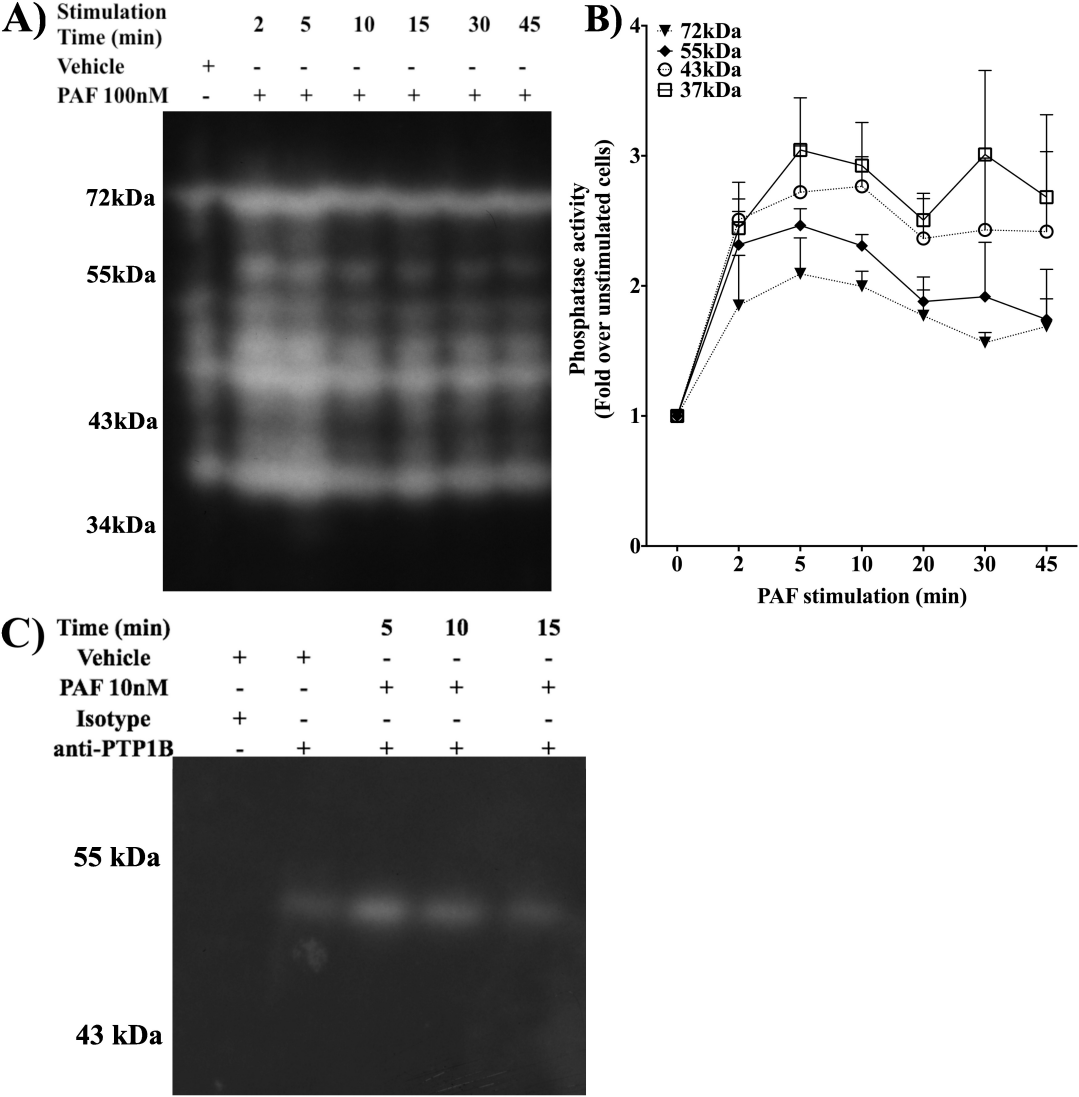
PAF induces PTP1B activity. **A)** Representative autoradiography with lysates of iMo-DCs stimulated with PAF. iMo-DCs (2x10^6^ cells) were starved for 3h in serum-free medium with 0.2% BSA and stimulated with 10nM PAF for indicated times. Cells were lysed with in-gel PTP lysis buffer and 15μg of whole cell lysate was loaded onto a 10% acrylamide SDS-PAGE gel, copolymerized with labeled poly(Glu-:Tyr). **B)** Graph summarizing the normalized intensity of bands according to the molecular weight and stimulation time. Data presented are mean ±S.E.M of normalized intensity (as described in Materials & Methods) for 3 independent experiments. **C)** Representative autoradiography obtained with PTP1B immunoprecipitated from Mo-DCs, stimulated with 10nM PAF for indicated times. Data are representative of at least 3 independent experiments.

Previous studies have shown that PAF stimulation can induce IL-6 production in a PLC- PLA_2_- [17] 5-lipoxygensase- [18] and calcium-dependent [17, 19] manner. Our laboratory has shown that PAF activates Jak2 and Tyk2 leading to STAT1, STAT2, STAT3 and STAT5 tyrosine phosphorylation [16, 31]. Others have shown that STAT1 and STAT5 could modulate IL-6 production either by directly binding to the promoter [29] or via induction [25] or interaction [27] with other transcription factors that bind the hIL-6 promoter [28,30]. Taking into consideration these data, we decided to investigate the role of the regulation of the Jak/STAT pathway in PAF-induced IL-6 production in immature Mo-DCs (iMo-DCs). Given the importance of PTPs, in particular PTP1B, in modulation of Jak2, Tyk2 and STAT activity [35], we first assessed whether PAF modulated PTP activity. Global representation of PAF-induced PTP activation was studied by in-gel PTP assays. iMo-DCs (**Fig 1A**) or HEK-PAFR (**S1 Fig**) were stimulated with PAF, for indicated times, and PTP activity was measured as described in Material and Methods. In both cell types, PAF induced an increase in PTP activity in a time-dependent manner. Compilation of normalized densities obtained from different donors shows that PAF stimulation increased the activity of several PTPs with molecular weights of approximately 55, 43 and 37kDa and more modestly, compared to unstimulated controls, ones with molecular weights of 72kDa. This PAF-stimulated phosphatase activity could be due to full length (50kDa) PTP1B and its cleaved segments (43 and 37kDa), in addition to activation of other PTPs. As we were interested in the modulation of the Jak/STAT pathway and PTP1B is a phosphatase associated with Jak2 modulation, we pursued this avenue.

We immunoprecipitated PTP1B from lysates of iMo-DCs stimulated with 10nM PAF. In-gel PTP assays showed that PTP1B activity was transiently increased in iMo-DCs 5 min post-stimulation with PAF and then decreased at 10 and 15 min (**Fig 1C**). Our data indicate that PAF could modulate PTP activation in different cell types in a time-dependent manner and that PTP1B is among the PTPs stimulated by PAF.

### PTP1B regulates PAF-induced IL-6 mRNA levels in DCs

Previous studies from our laboratory had demonstrated that PAF can be involved in polarization of lymphocytes toward a Th17 phenotype in a IL-6-dependent manner, via IL-6 production by Langerhans cells, a subtype of DCs [13]. We therefore studied the potential involvement of PTP1B in PAF-induced IL-6 mRNA levels in iMo-DCs by using iMo-DCs transfected with control (siCTRL) or PTP1B-specific (siPTP1B) siRNA.

**Fig 2:**
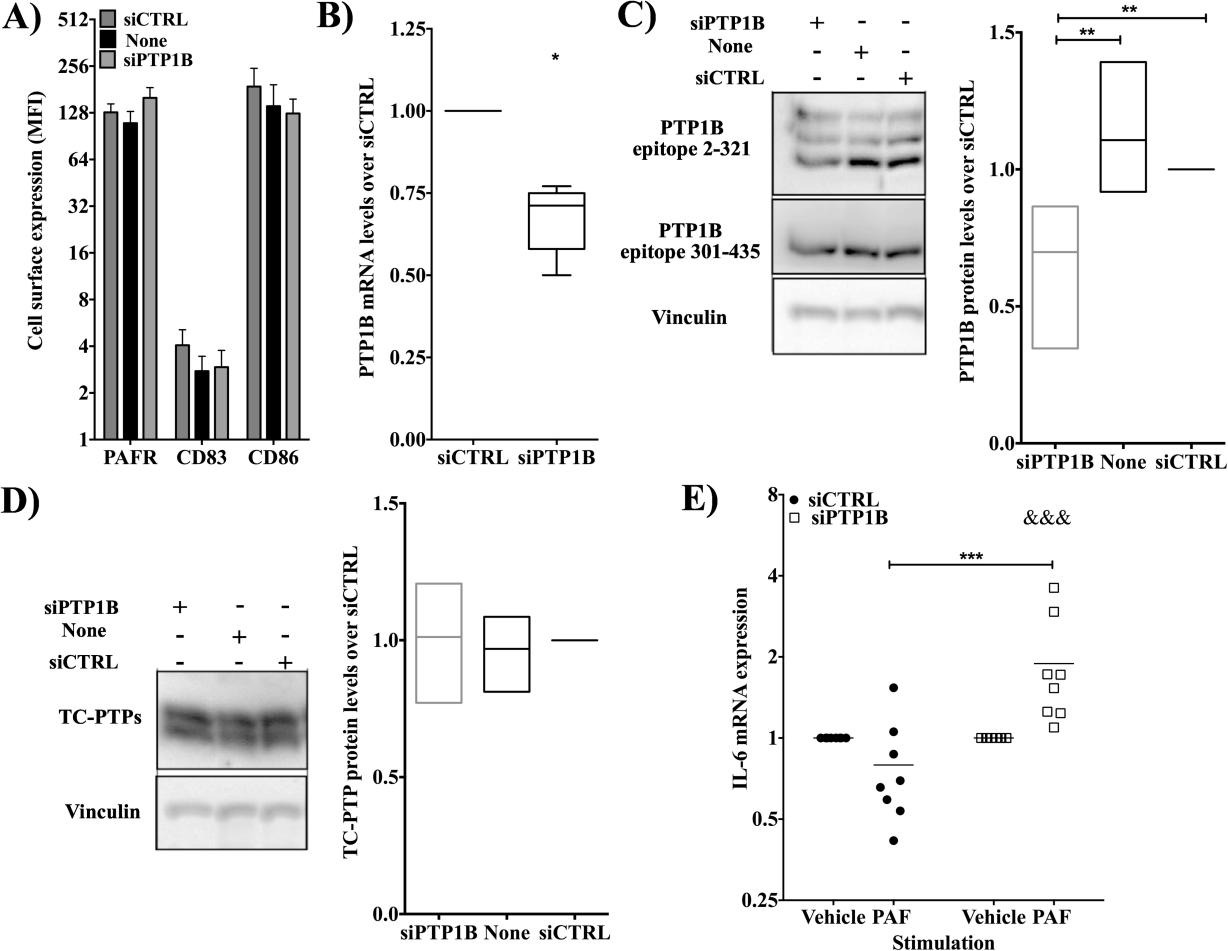
PTP1B regulates PAF-induced IL-6 mRNA levels in human immature Mo-DCs. iMo-DCs were transfected with siRNAs, either control (siCTRL) or against PTP1B (siPTP1B) at day 4 and 5 before being collected at day 7 for experiments. **A)** Mean fluorescence Intensity (MFI) of surface expression of CD83, CD86 and PAFR. At day 7, iMo-DCs, transfected with siCTRL or siPTP1B, were collected, fixed and stained with antibodies against indicated markers. Cell surface expression was analyzed on a FACSCalibur flow cytometer. Data presented are mean±S.E.M of 5 independent experiments **B)** iMo-DCs were lysed in Trizol and RNA was extracted and converted to cDNA, as described in Materials and Methods. GAPDH and PTP1B expression was measured using real-time PCR. Results are presented as 2^ΔΔCT^ relative to siCTRL. Data presented are in Box & Whisker graph (Min to Max) of 5 independent experiments. Significance was established with Student's t-test. **C)** Representative blot of 6 experiments performed: PTP1B levels in siCTRL and siPTP1B treated iMo-DCs. At day 7, iMo-DCs were lysed and whole cell lysates were loaded onto SDS-PAGE, transferred to a nitrocellulose membrane and blotted with goat anti-PTP1B, epitope 2–321, or mouse anti-PTP1B, epitope 301–435, antibodies. Blots were scanned and variations in PTP1B levels normalized with vinculin are represented in a graph as fold change over siCTLR cells. Data presented are in Floating bar (Min to Max) with line at the mean of 3–5 independent experiments. Significance was established with Kruskal-Wallis test with Dunn's multiple comparaison test. **C)** Representative blot of TC-PTP levels in untreated, siCTRL- or siPTP1B-treated iMo-DCs. TC-PTP levels were determined by blotting with an anti-TC-PTP antibody. Variations in TC-PTP levels normalized with vinculin are represented in a graph as fold change over siCTLR cells. Data presented are in Floating bar (Min to Max) with line at the mean of 3–5 independent experiments. **D)** iMo-DCs, transfected with siCTRL or siPTP1B were stimulated for 5hrs with vehicle or PAF (10nM), then lysed in Trizol and RNA was extracted and converted to cDNA. GAPDH and IL-6 expression was measured using real-time PCR. Results are presented as 2^ΔΔCT^relative to unstimulated cells. Data presented are mean±S.E.M of 6 independent experiments. Significance was established with paired two-way anova with Sidak post-test.: *p<0.05, ***p<0.01 and &:p< 0.05 over unstimulated cells with the same pre-treatment.

First, we ensured that the siRNAs did not activate the iMo-DCs or induce maturation, both associated with a decrease in PAFR expression [47] and with induction of different pathways that could affect the interpretation of our results. Cell surface expression of CD83, a maturation marker, CD86, a differentiation marker, and PAFR was not significantly affected by transfection with siCTRL or siPTP1B (**Fig 2A**), confirming that any difference in results between the two populations was not due to a difference in the cell activation state or PAFR expression. Secondly, we tested the efficiency and specificity of these siRNA. siRNAs against PTP1B caused a 30% decrease in PTP1B mRNA (**Fig 2B**) and protein (**Fig 2C**) levels compared to siCTRL. In addition, the closely related phosphatase TC-PTP was not modulated by the siRNAs we used (**Fig 2D**).

When PAF-induced IL-6 mRNA levels were measured in siRNA-transfected iMo-DCs, a significant increase of IL-6 mRNA levels was observed in iMo-DCs transfected with siPTP1B compared to siCTRL-transfected cells (**Fig 2E**). These results indicate that PAF-induced IL-6 mRNA expression in iMo-DCs was regulated by PTP1B.

### PTP1B regulates PAF-induced hIL-6 promoter activation in a Jak2-dependent manner

Previous studies from our group have demonstrated that Tyk2 and Jak2 are activated by PAFR stimulation, whereas others have shown that PTP1B dephosphorylates both these kinases [16, 31, 35]. Our previous data had also indicated that Jak2 activation by PAFR may result in negative regulation of PAFR signaling [31]. We therefore concentrated on this kinase in conjunction with PTP1B activation. We first examined whether PTP1B could modulate PAF-induced IL-6 promoter activation in HEK-PAFR and whether Jak2 had a role in this modulation.

**Fig 3:**
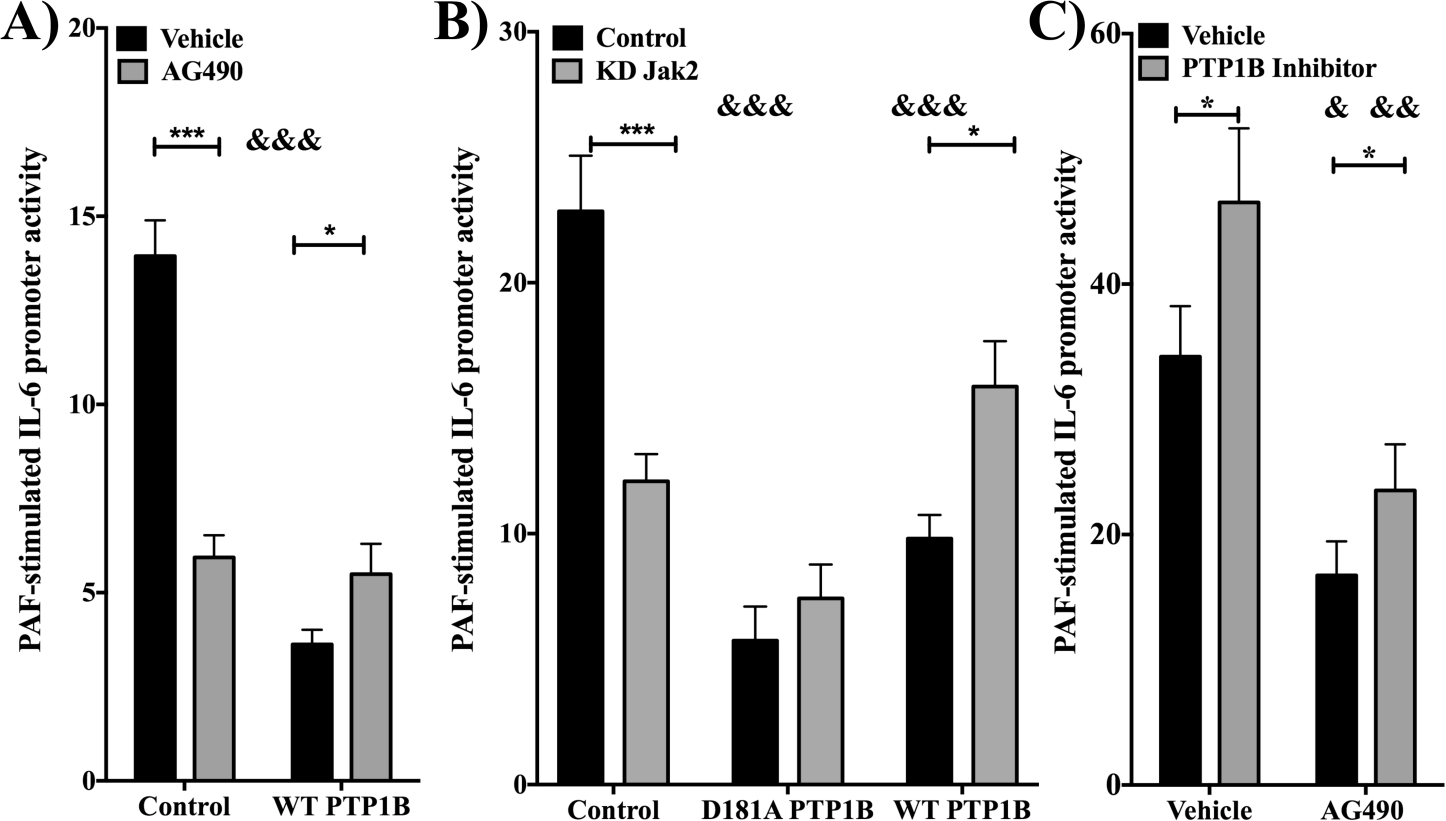
PTP1B regulates PAF-induced hIL-6 modulation in a Jak2-dependent manner. **A)** HEK-PAFR were transiently co-transfected with hIL-6 promoter luciferase constructs and PTP1B or vector control (pcDNA-3). After starvation, cells were pre-treated for 20min with 5μM AG490 before a 6h stimulation with PAF (100nM) or vehicle. **B)** HEK-PAFR were transiently co-transfected with hIL-6 promoter/luciferase construct and WT or D181A mutant PTP1B, and/or kinase-dead mutant Jak2 or control vectors. After starvation, cells were stimulated for 6h with PAF (100nM) or vehicle (EtOH) and IL-6 promoter activity was established. **C)** HEK-PAFR were transfected with hIL-6 promoter/luciferase construct and vector control. After starvation, cells were pre-treated for 20min with 4μM PTP1B inhibitor before a 6h stimulation with PAF (100nM) or vehicle and IL-6 promoter activity was determined. For all luciferase assays, results are presented as fold induction of IL-6 promoter activity induced by PAF over vehicle control. The data presented are mean±S.E.M of at least 4 experiments performed in triplicate. Significance was established with paired two-way anova with Sidak post-test. *: p<0.05; ***:p<0.001 and &: p<0.05 &&&: p<0.001 compared to conditions without overexpression or inhibition of PTP1B.

**Fig 3A** and **3B** show, first, that Jak2 inhibition alone, by use of the pharmacological inhibitor AG490, or transfection of a dominant negative Jak2 (KD Jak2) caused a decrease in PAF-induced IL-6 promoter activity. Secondly, as found in iMo-DCs, PTP1B negatively modulated PAF-induced IL-6 promoter activity. In addition, both Jak2 dominant negative mutant and AG490 partially, but significantly, reversed the inhibition by PTP1B of PAF-stimulated IL-6 transcription, indicating that Jak2 had a role in PTP phosphatase activity, since the decrease caused by the transfection of a phosphatase inactive mutant (D181A PTP1B) was not reversed by the KD Jak2. This indicates that the increase caused by co-transfection of the KD Jak2 in PTP1B-transfected cells needs a catalytically active form of PTP1B. This suggests that Jak2 could be upstream of PTP1B and that Jak2 may have a dual function in PAF-stimulated IL-6 mRNA expression.

To confirm that the effect of PTP1B seen on PAF-induced hIL-6 activation depended on phosphatase activity and was not only due to overexpression, luciferase assays were performed using PTP1B inhibitor as pre-treatment, combined with AG490 or its vehicle (**Fig 3C**). As expected, inhibition of PTP1B increased PAF-induced hIL-6 promoter activation. AG490 significantly decreased PAF-induced promoter activity in vehicle and PTP1B inhibitor pre-treated cells, suggesting that Jak2 was needed for a maximal transduction in both cases. Moreover, when cells treated with AG490 were co-incubated with the PTP1B inhibitor, the PAF-induced hIL-6 promoter activity was partially rescued, suggesting that the decrease caused by AG490 depended to some extent on PTP1B activity. These results suggest that Jak2 kinase activity is important for PAF-induced transactivation of hIL-6 promoter, that PTP1B regulates this transactivation in a phosphatase-dependent manner and that this modulation partly depends on Jak2 activity that could be upstream of PTP1B activity.

**Fig 4:**
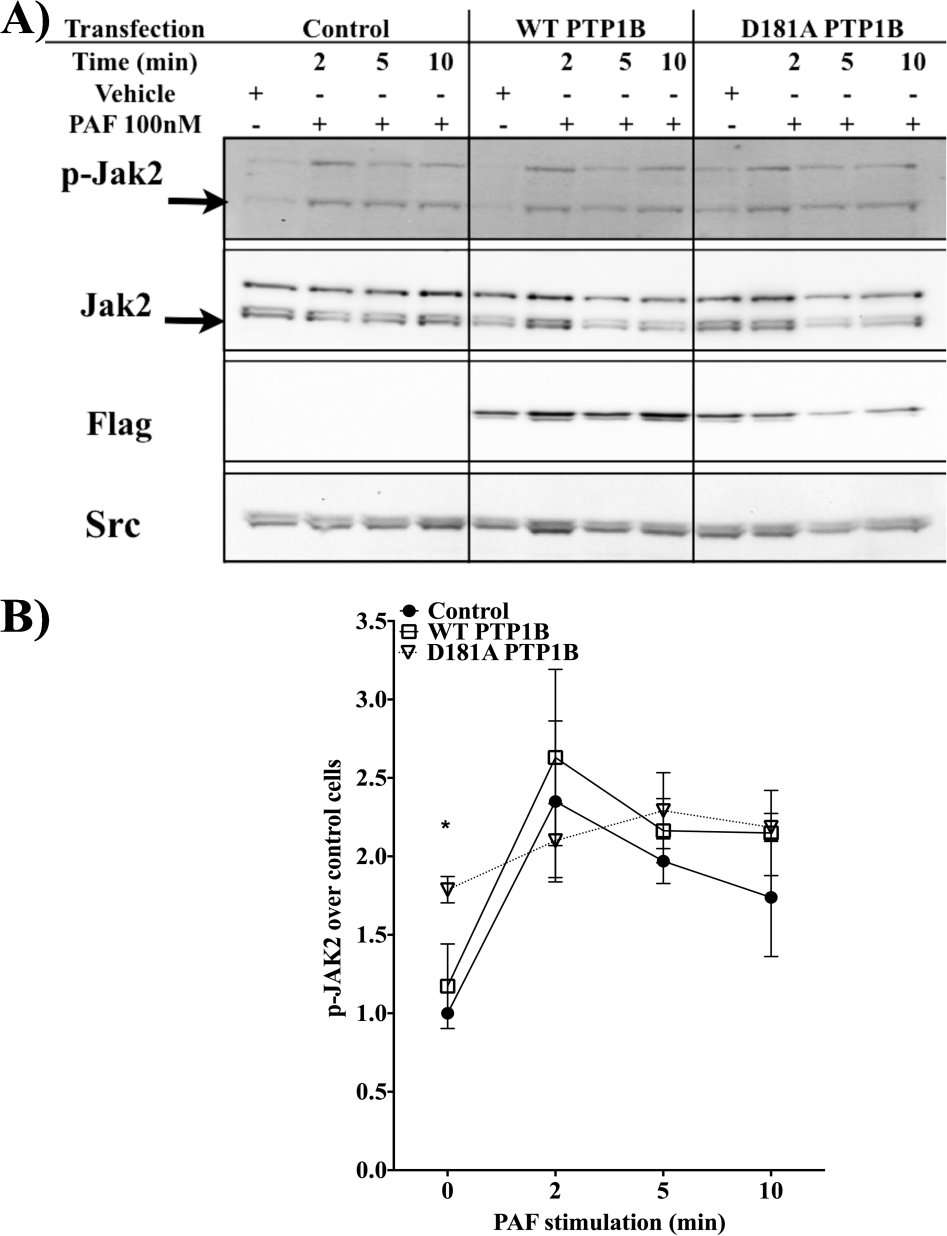
PTP1B does not modulate PAF-induced Jak2 phosphorylation. **A)** Representative blot of experiments conducted with HEK-PAFR transfected with Flag-tagged wt or D181A PTP1B or control vector for 8h before overnight starvation in DMEM, 0.2% BSA. Cells were stimulated for indicated times with PAF (100nM) or vehicle. Cells were lysed and western blots were performed. **B)** Blots were scanned and the variation in Jak2 phosphorylation (pJak2 Tyr 1007/1008) is represented as ratio of phosphorylation in stimulated cells over control unstimulated cells. Data presented are mean±S.E.M of 5 independent experiments. Significance was established with paired two-way anova with Sidak post-test. *: p<0.05.

To test this hypothesis, we therefore investigated whether PTP1B could modulate Jak2 activation. We examined whether PTP1B had an effect on Jak2 phosporylation in PAF-stimulated cells using HEK-PAFR cells transfected with control vector or WT PTP1B. As shown in **Fig 4A**, PAF induced a rapid phosphorylation of Jak2, within 2 min post-stimulation, which then decreased after 5 min. A compilation of 5 experiments, **Fig 4B,** shows that mutant PTP1B increases phosphorylation levels of Jak2 in unstimulated cells, indicating that PTP1B could be involved regulation of basal activation state of this kinase. On the other hand, transfection of WT or D181A PTP1B did not significantly alter the PAF-induced phosphorylation levels of Jak2 after PAF stimulation. These results suggest that PTP1B does not participate in PAF-induced Jak2 activation, indicating that, possibly, it may be Jak2 which had a role in modulating PTP1B activity.

**Fig 5:**
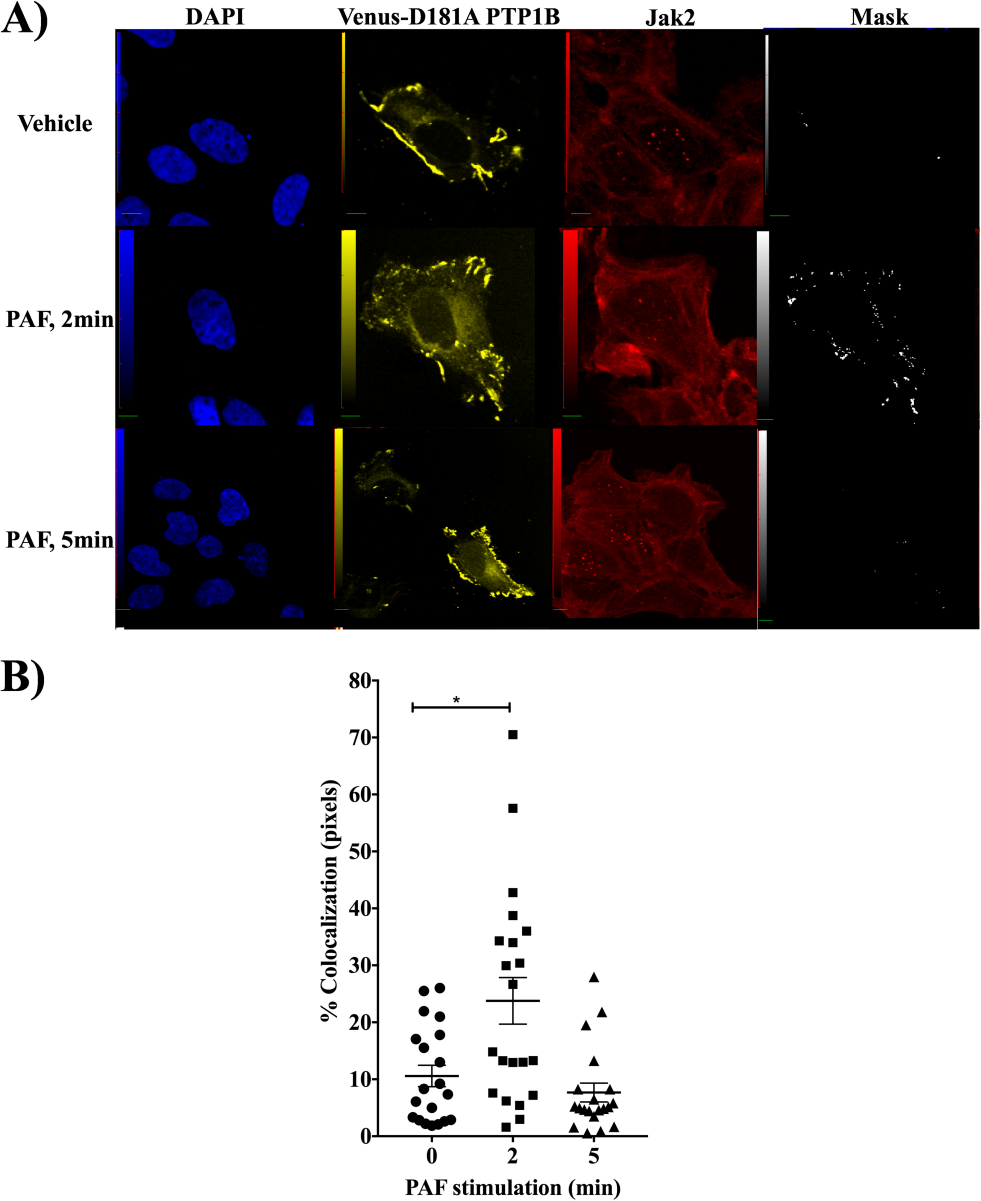
PTP1B is found in the same complex as Jak2. **A)** HEK-PAFR, grown on poly-L-lysine coated coverslips, were transfected with Venus-tagged D181A-PTP1B then stimulated, 30h later, with vehicle or with 100nM PAF for 2 or 5min before permeabilization with 0,5% Triton X-100. The cells were stained overnight with rabbit anti-Jak2 antibodies or control isotype (not shown), followed by anti-rabbit-Cy5 staining. Nuclei were stained with DAPI and cells were analyzed by confocal microscopy. Results are representative of 4 independent experiments, with at least 4 pictures from different fields in each experiment. Scale bar = 5μm. **B)** Variation of colocalization is presented as % of colocalizated pixels at different time points where each dot represents a cell. Data presented are mean ±S.E.M of 4 independent experiments. Significance was established with one-way anova, Tukey's Multiple Comparison post-test *: p<0.05.

We then investigated whether the two molecules could co-localize, either in the basal state or after PAF stimulation. Using HEK-PAFR cells transfected with the Venus-tagged substrate-trapping mutant D181 PTP1B and Abs agains Jak2, we found a low level of PTP1B and Jak2 co-localization in the basal state, but after PAF stimulation, there was a rapid and transient increase in co-localized pixels at 2 min, which was no longer visible after 5 min of stimulation (**Fig 5A & 5B**). Given that we used a trapping mutant which should not release its substrate, if bound by the PTP domain, this suggests, in agreeement with results obtained in Fig 4 that Jak2 and PTP1B may interact, either through other domains than the PTP domain or indirectly, via other proteins. Western blot experiments combined with microscopy suggest that PTP1B does not directly regulate Jak2 activation but is found in the same transitory signaling complexes. Maximal down-regulation of PAF-induced IL-6 promoter activity by PTP1B seems to depends on Jak2, suggesting that PTP1B acts downstream of Jak2.

**Fig 6:**
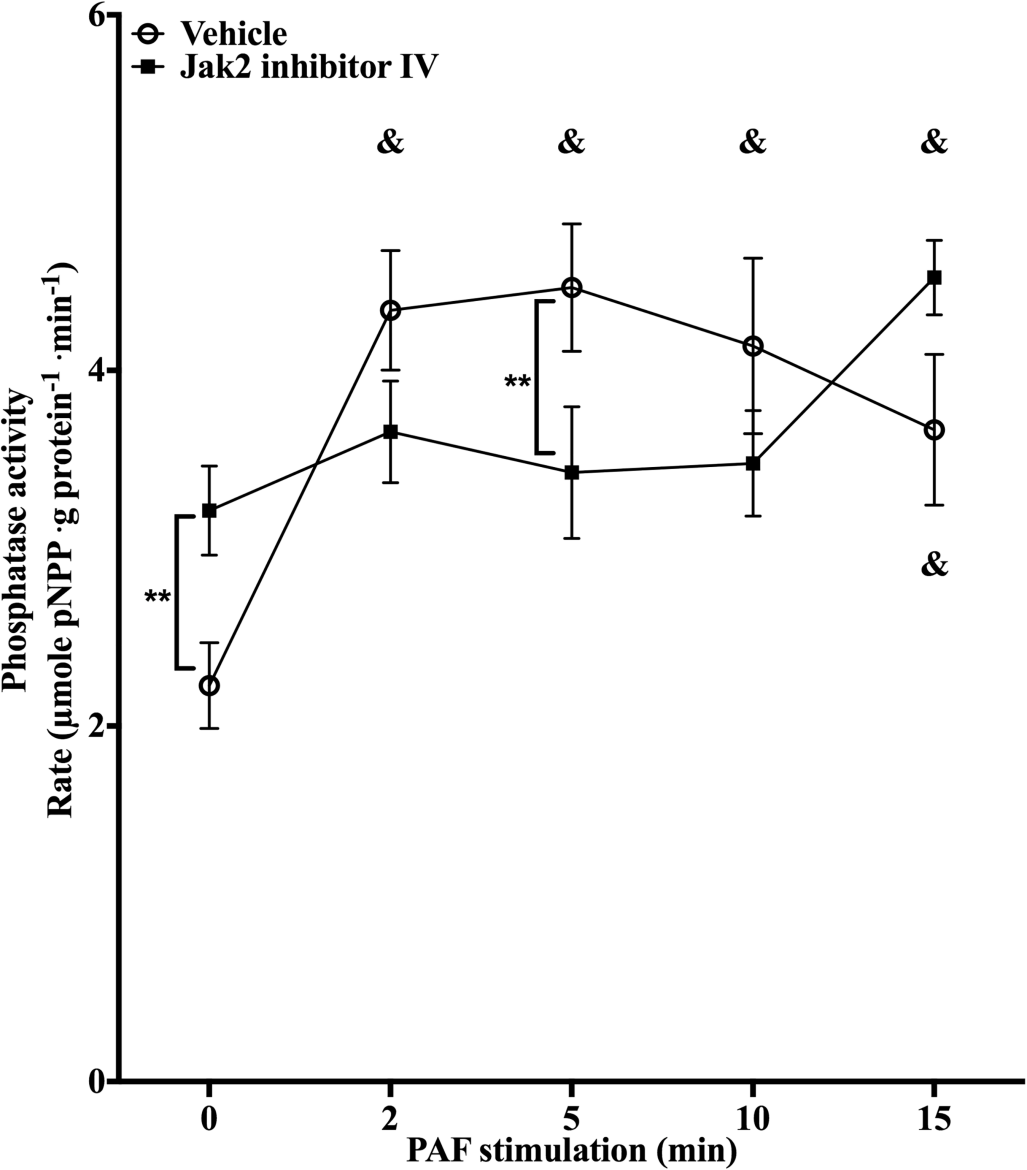
Jak2 is involved in PAF-induced PTP1B activity. Graphs represent hydrolysis rate of pNPP by PTP1B immunoprecipitated from HEK-PAFR stimulated with 100nM PAF for indicated times, after pre-treatment with 2μM Jak2 inhibitor IV. PTP1B was immuoprecipitated from HEK-PAFR, which had been starved overnight in DMEM 0.2% BSA, pre-treated and stimulated, as indicated, before being incubated with pNPP. Data presented are mean ±S.E.M of pNPP hydrolysis rate for at least 3 independent experiments. Significance was established with paired two-way anova with Sidak post-test. *p<0.05, ** p<0.01 and &:p< 0.05 over unstimulated cells with the same pre-treatment.

Next, the involvement of Jak2 in PAF-induced PTP1B activation was tested by colorimetric PTP assays using pNPP as substrate. PTP1B was immunoprecipitated from HEK-PAFR which had been pre-treated for 30min with 2μM of Jak2 inhibitor IV and then stimulated with 100nM PAF for indicated times. **Fig 6** shows that Jak2 inhibitor significantly reduced PAF-induced PTP activation. Effectively, PAF induced a significant increase in PTP1B activity and this, as soon as 2 min post-stimulation in control cells and activity remained significantly higher than basal state for 10min. In Jak2 inhibitor IV-pre-treated cells, PAF did not induce a significant increase in PTP1B activity, compared to basal activity in unstimulated cells. Even if basal levels of pre-treated cells were higher than those found in control cells, the pNPP hydrolysis rate of PTP1B in Jak2 inhibitor IV-pre-treated cells was slightly lower (p = 0.16) at 2min and significantly lower at 5min post-stimulation, than the rate in vehicle pre-treated cells stimulated with PAF for the same time periods. Results obtained with Jak2 inhibitor IV were confirmed using 200nM SD-1029 (another Jak2 inhibitor) as pretreatment for HEK-PAFR stimulated with PAF for 5 or 10min (**S2 Fig**). These data suggest that Jak2 is involved in the activation of PTP1B during PAF stimulation in a kinase-dependent manner, and also suggest the involvement of Jak2 in regulating the basal activity of this phosphatase.

### Jak2 regulates PTP1B cleavage

**Fig 7:**
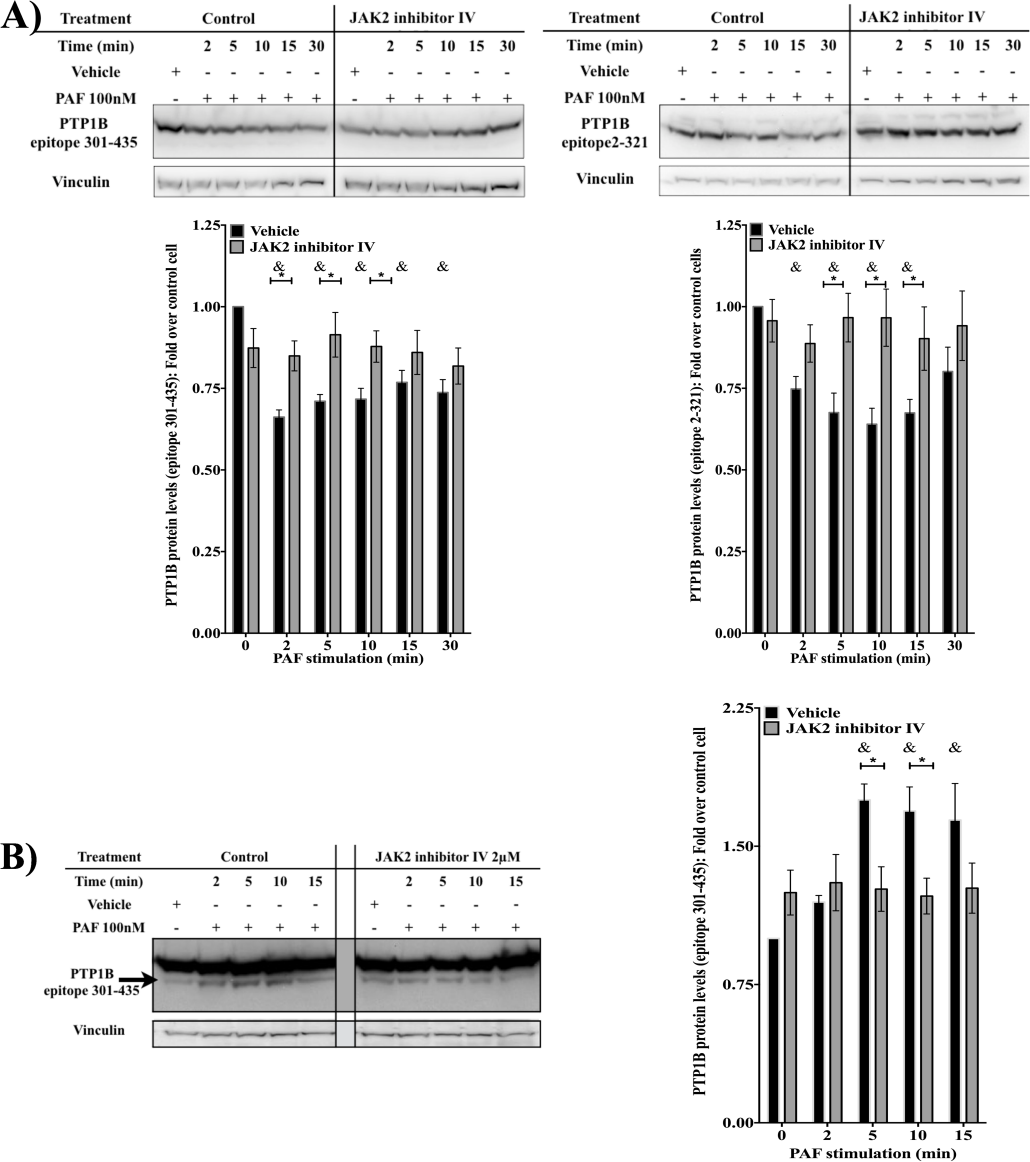
Jak2 regulates PTP1B cleavage. HEK-PAFR were starved overnight and pre-treated with vehicle or 2μM Jak2 inhibitor IV for 20min prior to stimulation with 100nM PAF, for indicated times. Whole cell lysates were loaded onto SDS-PAGE, transferred to nitrocellulose membranes and blotted overnight with indicated antibodies: mouse anti-PTP1B (epitope 301–435) or goat anti-PTP1B (epitope 2–321) and PTP1B expression was followed over time. **A)** Representative western blots of PTP1B levels are shown and in the lower panels data are presented as relative intensity of PTP1B expression over vinculin and reported as fold change, at indicated times. **B)** Representative western blot of PTP1B levels, that had been over-exposed in order to show the cleaved fragment. In the right panel data are presented as relative intensity of the cleaved PTP1B fragment, over vinculin expression and reported as fold change at indicated times. Data presented are mean±S.E.M. of at least 5 experiments. Significance was established with paired two-way anova with Sidak post-test: *p<0.05; &p<0.05 compared to unstimulated cells.

It has been shown that Jak2 and calpain could be involved in the same signaling pathways [48]. Given that calpain is also known to modulate PTP1B activity [49–51], we investigated whether Jak2 modulated PAF-induced PTP1B activity in a calpain-dependent manner. First, we tested whether PAF could induce cleavage of PTP1B and whether Jak2 could be involved in this cleavage. This question was addressed by pre-treating HEK-PAFR for 20min with 2μM Jak2 inhibitor IV, or vehicle, and stimulating the cells with 100nM PAF, for indicated times. Western blots were performed with 2 different antibodies directed against 2 different regions of PTP1B, the sequences encompassing the cleavage site of oxidized PTP1B between Glu76-Ala 77 and the putative cleavage site of unoxidized PTP1B found between residues 360–381 [50–51]. In **Fig 7A**, we show that, for both antibodies, PAF induced a decrease in the major band, found around 50kDa and this, in a time-dependent manner, indicating that cleavage had occurred. A decrease can be observed as soon as 2 min post-stimulation with a significant decrease at 5 min. This decrease is maintained until 30 min post stimulation. The PTP1B levels, for the 50kDa band, were significantly higher in Jak2 inhibitor IV-treated cells at 5 and 10 min post-stimulation compared to control cells and this for both antibodies (**Fig 7A**). Same results were obtained in SD-1029 pre-treated cells stimulated 5 or 10 min with 100nM PAF (**S3 Fig**). When more lysate was loaded on the gel, a band under the 50kDa major band was detected with the antibody against the C-terminal part of PTP1B (**Fig 7B**). This shorter isoform was faintly detected under basal conditions in control cells, increased in expression within 2 min after stimulation, and was significantly increased after 5 min of stimulation. The elevated levels were maintained for all tested times. In Jak2 inhibitor pre-treated cells, this isoform of PTP1B was slightly higher at basal levels than in control cells but the PAF-induced increase was not seen. The increase in detected levels for this isoform correlated with the decreased levels for the 50kDa band in control cells (Spearman r = -0,4212, P = 0,0454) but not in Jak2 inhibitor pre-treated cells (Spearman r = -0.2731 P = 0.2579), suggesting that Jak2 could be important in the generation of this isoform, which seems to be the 42kDa PTP1B fragment generated by calpain described by other groups [49–51]. Since the decrease in the 50kDa band correlated with the appearance of the smaller fragment, for the rest of this article, the cleavage of PTP1B will be followed by the decrease of the 50kDa band considering that this decrease is more easily detected by both the antibodies used. Results obtained, here, suggest that PAF induces PTP1B cleavage in a Jak2-dependent manner.

**Fig 8:**
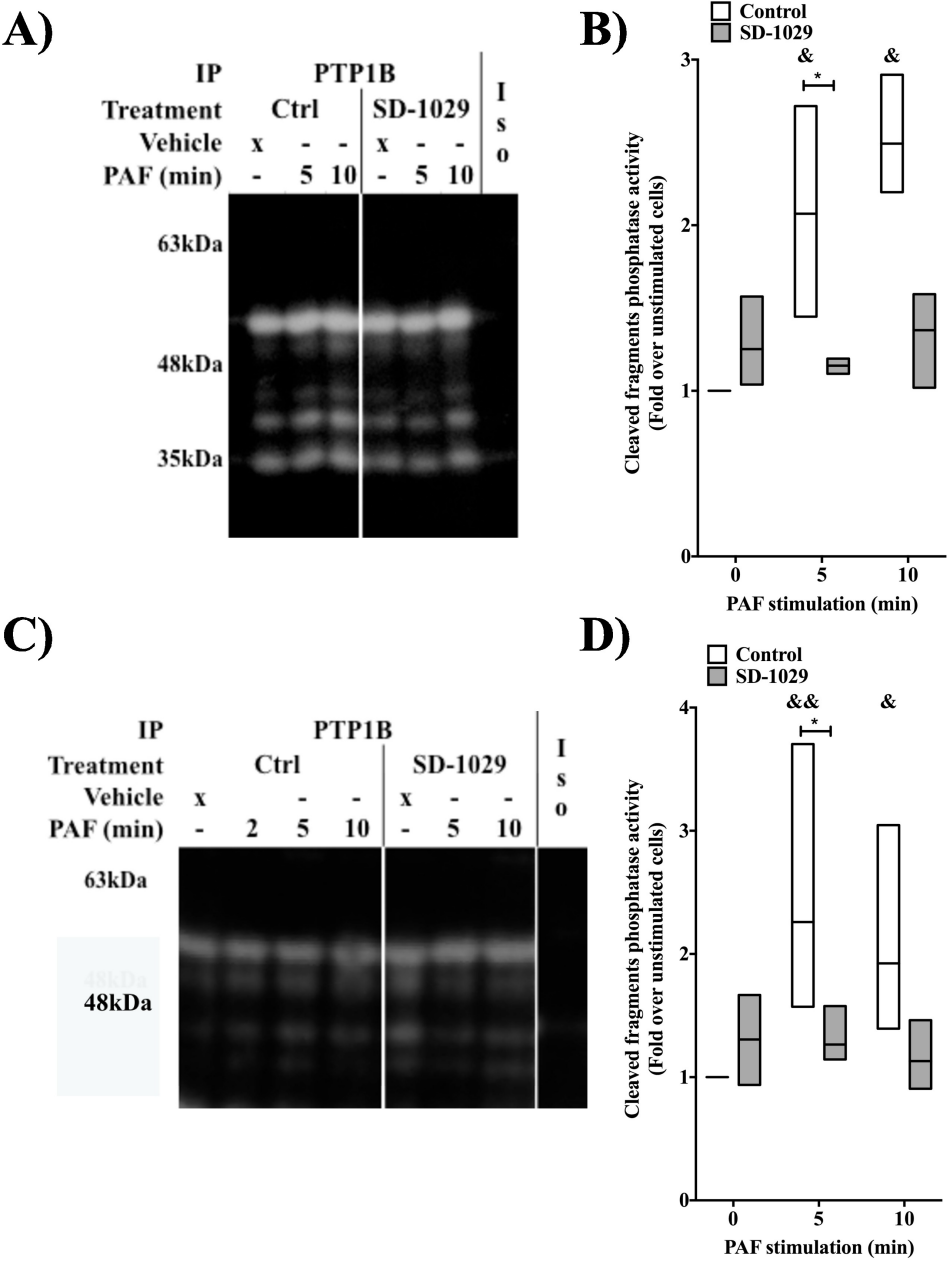
Jak2 regulates the activity of the cleaved fragments of PTP1B. A) Representative autoradiography obtained with PTP1B, immunoprecipitated from HEK-PAFR, stimulated with 100nM PAF for indicated times, after a 20 min pre-treatment with 200nMSD-1029 or its vehicle. Data are representative of 3 independent experiments. B) Graph summarizing the normalized intensity of bands with a molecular weight lower than 50 kDa and stimulation times. Data presented are mean±S.E.M. of normalized intensity (as described in Materials & Methods) for 3 independent experiments. C) Representative autoradiography obtained with PTP1B, immunoprecipitated from Mo-CD, stimulated with 10nM PAF for indicated times, after a 20 min pre-treatment with 200nMSD-1029 or its vehicle. Data are representative of 4 independent experiments. D) Graph summarizing the normalized intensity of bands with a molecular weight lower than 50 kDa and stimulation times. Data presented are mean±S.E.M. of normalized intensity (as described in Materials & Methods) for 4 independent experiments.

Next we determined if these cleaved fragments could indeed have a functional role by performing in-gel phosphatase assays with PTP1B immunoprecipitated from HEK-PAFR (**Fig 8A & 8B**) or Mo-DC lysates (**Fig 8C & 8D**). In order to clearly detect the fragments, we loaded more proteins (30ug of total protein against 15ug for **Fig 1C**) and thus the full length PTP1B activity is satturated and no modulation is visible. For bands with a molecular weight lower than 50 kDa, in both cell types, PAF induced a significant increase in PTP activity, measured after stimulation of 5 and 10 min. This increase was singnificantly lower when cells were pre-treated with the Jak2 inhibitor, SD-1029. These results that the cleaved fragments, observed after PAF stimulation, increase in phosphatase activity and could have a functional importance in PAF-induced signaling pathways. Since calpain has been found to be both a positive and negative regulator of PTP1B activity [49–51], we decided to further investigate the involvement of this protease in PAF-induced IL-6 production and in PAF-induced PTP1B activation.

### PAF regulates PTP1B activity in a calpain-dependent manner

**Fig 9:**
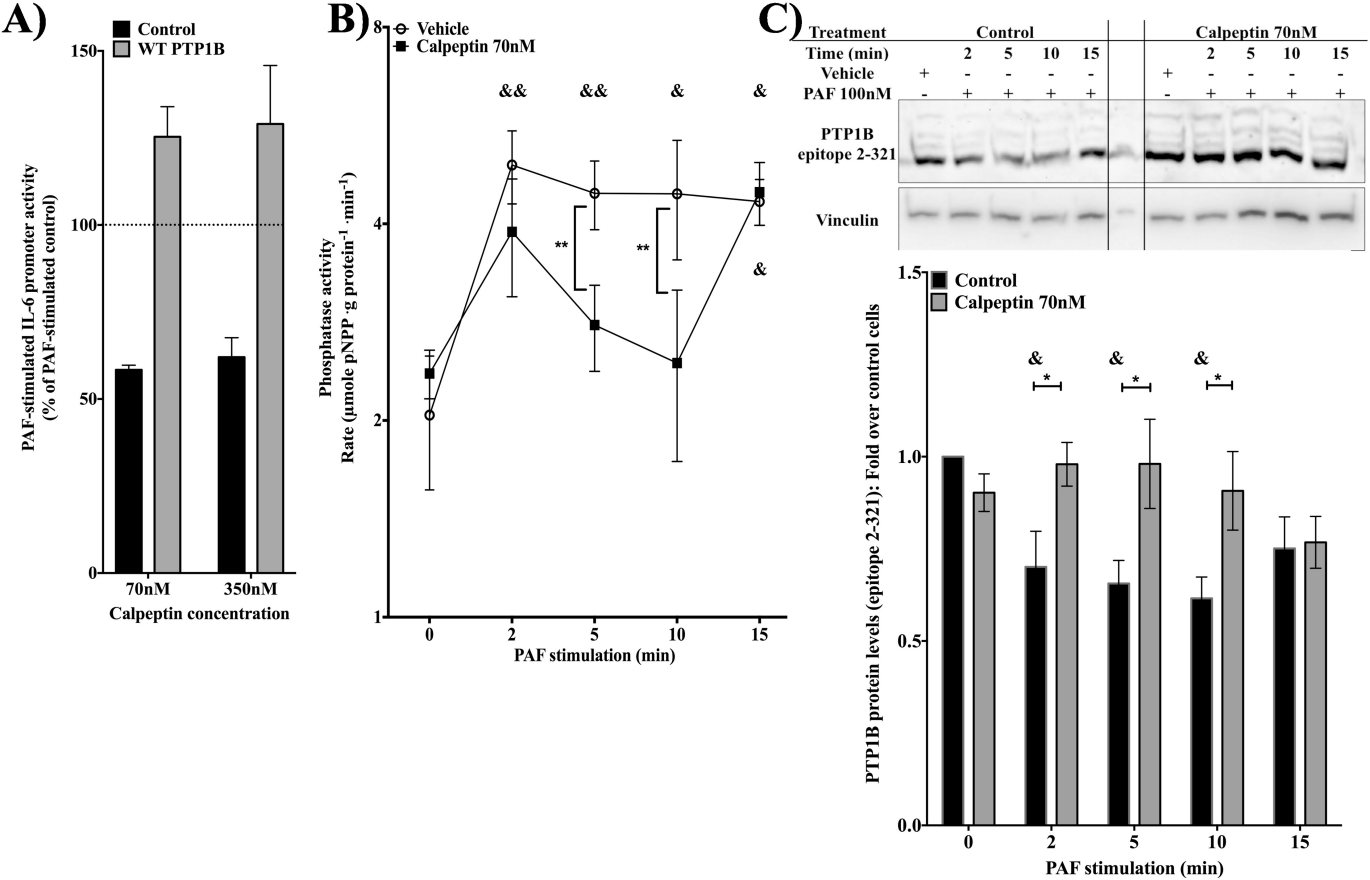
PAF regulates PTP1B activity in a calpain-dependent manner. **A)** HEK-PAFR were transiently co-transfected with hIL-6 promoter luciferase constructs, WT PTP1B or control vectors for 8h before overnight starvation in DMEM 0.2% BSA. After a 20min pre-treatment with 70 or 350nM calpeptin or vehicle, cells were stimulated for 6h with PAF (100nM) or vehicle and luciferase activity was measured. Luciferase activity of cells transfected with the control vector or WT PTP1B, pre-treated with the vehicle for calpeptin and stimulated with PAF was set at 100% and compared with the activity of cells treated with the two concentrations of calpeptin. The data presented are mean±S.E.M of at least 3 independent experiments performed in triplicate. Significance was established with paired two-way anova with Sidak post-test. &: p<0.05 &&: p<0.01. **B)** Hydrolysis rate of pNPP by PTP1B immunoprecipitated from HEK-PAFR stimulated with 100nM PAF for indicated times after pre-treatment with 70nM calpeptine or its vehicle. PTP1B was immunoprecipitated from HEK-PAFR, which had been starved overnight in DMEM 0.2% BSA, pre-treated and stimulated as indicated. Data presented are mean ±S.E.M of hydrolysis rate of pNPP for at least 3 experiments. Significance was established with paired two-way anova with Sidak post-test: ** p<0.01 and & p< 0.05 && <0.01 over unstimulated cells with the same pre-treatment. **C)** Representative western blot of PTP1B levels. HEK-PAFR were starved overnight, then pre-treated with 70nM calpeptin or vehicle for 20min prior to stimulation with 100nM PAF for indicated times. Whole cell lysates were loaded onto SDS-PAGE, transferred onto nitrocellulose membrane and blotted overnight with goat anti-PTP1B, epitope 2–321. In the lower panel data are presented as relative intensity of PTP1B expression over vinculin and reported as fold change at indicated times. Data presented are mean±S.E.M of 6 independent experiments. Significance was established with paired two-way anova with Sidak post-test: *p<0.05, &:p< 0.05 over unstimulated cells with the same pre-treatment.

First, the involvement of calpain in PAF-induced hIL6-promoter activation and in PTP1B-mediated down-regulation of the promoter was tested by luciferase assays. HEK-PAFR were transfected with WT PTP1B or control vector and pre-treated 20 min with vehicle or calpeptine, a calpain inhibitor, and stimulated or not with 100nM PAF. Calpeptine, at both concentrations used, decreased PAF-induced hIL-6 promoter activity to 63,3±11.1% and 58.0±5.2% of activity in control cells (**Fig 9A**). In PTP1B-transfected cells, the PAF-induced promoter activity reached 51.0±8.9% of hIL-6 promoter activity obtained in control vector-transfected cells. When PTP1B-transfected cells were pre-treated with calpeptine, the PAF-induced hIL-6 promoter activation was partially rescued, since the promoter activity resembled that of calpeptine-treated cells in the absence of PTP1B overexpression (62.8±7.4%, at 350nM calpeptine, and 63.5±10.8%, at 70nM, of the PAF-induced hIL-6 promoter activity observed in control cells). This was an increase of around 25% when compared to untreated PTP1B-transfected cells. This suggests that, in HEK-PAFR stimulated with PAF, calpain is involved in maximal transactivation of the IL-6 promoter but is also important for maximal down-regulation hIL-6 promoter by PTP1B.

Next, we tested whether calpain is involved in PAF-induced PTP1B activation. Colorimetric assays of PTP1B activity were performed with PTP1B immunoprecipitated from HEK-PAFR pre-treated with calpeptin or with vehicle and stimulated with PAF for indicated times. Calpeptin inhibited, in a time-dependent manner, PAF-induced PTP1B activation (**Fig 9B**). In the cells pre-treated with the inhibitor, PTP1B activity was at 67.8±25.4% (at 5 min) and 54.18±14.6% (at 10 min) of control cells. To ensure that activation of PTP1B could be correlated to cleavage of PTP1B by calpain, PTP1B levels were measured by western blot. Lysates from HEK-PAFR pre-treated with calpeptin or vehicle and stimulated with PAF, for different times, were studied. In control cells, levels of PTP1B detected after stimulation were significantly lower than those in unstimulated cells (**Fig 9C**). However, in cells stimulated with PAF, PTP1B levels detected in calpeptin pre-treated cells were approximately 40% higher at 5 min and 45% higher at 10 min, compared to control cells. Similar results were obtained with anti-PTP1B directed against C-terminal tail (**S4 Fig**). Thus, results indicate that PAF induced a decrease in PTP1B 50kDa levels and this decrease was abrogated by the calpain inhibitor. Together, these results suggest that, by activating calpain, PAF induces a cleavage of PTP1B and an increase in its phosphatase activity, engendering a negative feedback for hIL-6 promoter activation. Next, we investigated whether the PAF-induced calpain activation could be under control of Jak2, given that inhibition of this kinase also inhibits the cleavage of PTP1B, as does inhibition of calpain.

### Jak2 regulates PTP1B activity in a calpain-dependent manner

**Fig 10:**
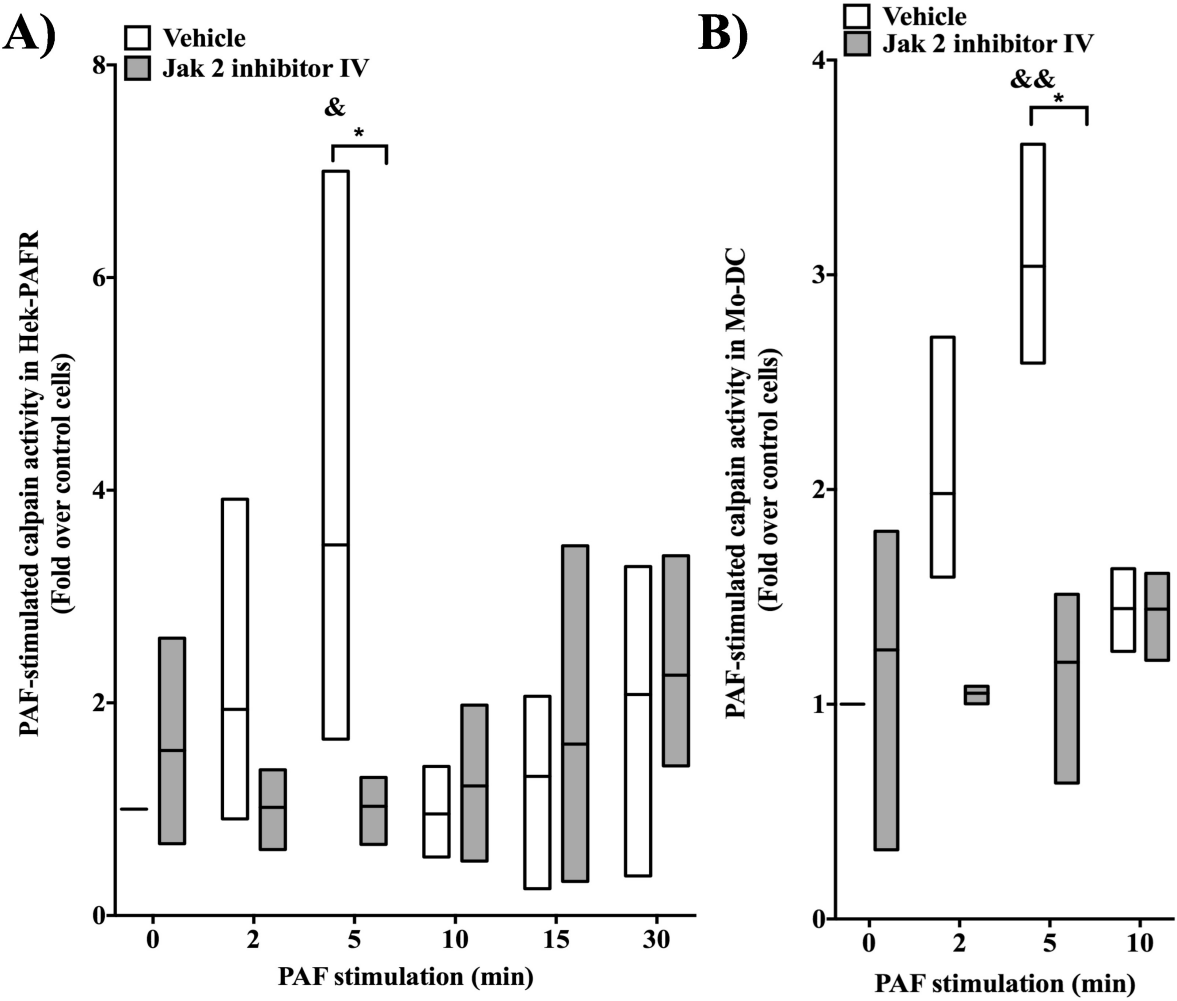
Jak2 is involved PAF-induced calpain activity. HEK-PAFR were starved overnight **A)** or **B)** Mo-DCs were starved for 5h, then pre-treated with vehicle or 2μM Jak2 inhibitor IV for 20min prior to stimulation with PAF for indicated times and lysis with Fluorometric Activity Assay Kit lysis buffer. Assays were performed according to manufacturer's instructions. Variations in calpain activity levels are presented as ratio of fluorescence of stimulated cells over untreated, unstimulated cells. Data presented are in Floating bar (Min to Max) with line at the mean of 3–4 independent experiments, for HEK-PAFR and 3 different donors, for iMo-DCs. Significance was established with paired two-way anova with Sidak post-test.*p<0.05, &:p< 0.05, &&: p<0.01 over unstimulated cells with the same pre-treatment.

To explore the possibility that Jak2 modulates calpain activity, calpain assays using a fluorescent substrate were performed. HEK-PAFR were pre-treated with Jak2 inhibitor IV (**Fig 10A**) or SD-1029 (**S5 Fig**) for 20min before stimulation with 100nM PAF for indicated times. As seen in **Figs 10A** and **S5**, PAF transiently activated calpain activity with a peak at 5 min post-stimulation and a return to basal activity at 10 min. Pre-treatment with either of the inhibitors abrogated PAF-mediated calpain activation indicating a role of Jak2 in PAF-induced activation of calpain at early stimulation times. Given that PAF activates PTP1B in iMo-DCs, involvement of Jak2 in calpain activation was also investigated in these cells. iMo-DCs were incubated for 20 min with Jak2 inhibitor IV or vehicle, then stimulated with PAF for indicated times **(Fig 10B**). A rapid and transient increase in calpain activity, with a peak at 5 min post-stimulation, was observed in PAF-stimulated control cells, while no significant variation in calpain activity was found when cells were pre-incubated with the Jak2 inhibitor.

### Jak2 regulates PTP1B in a calcium-dependent manner

**Fig 11:**
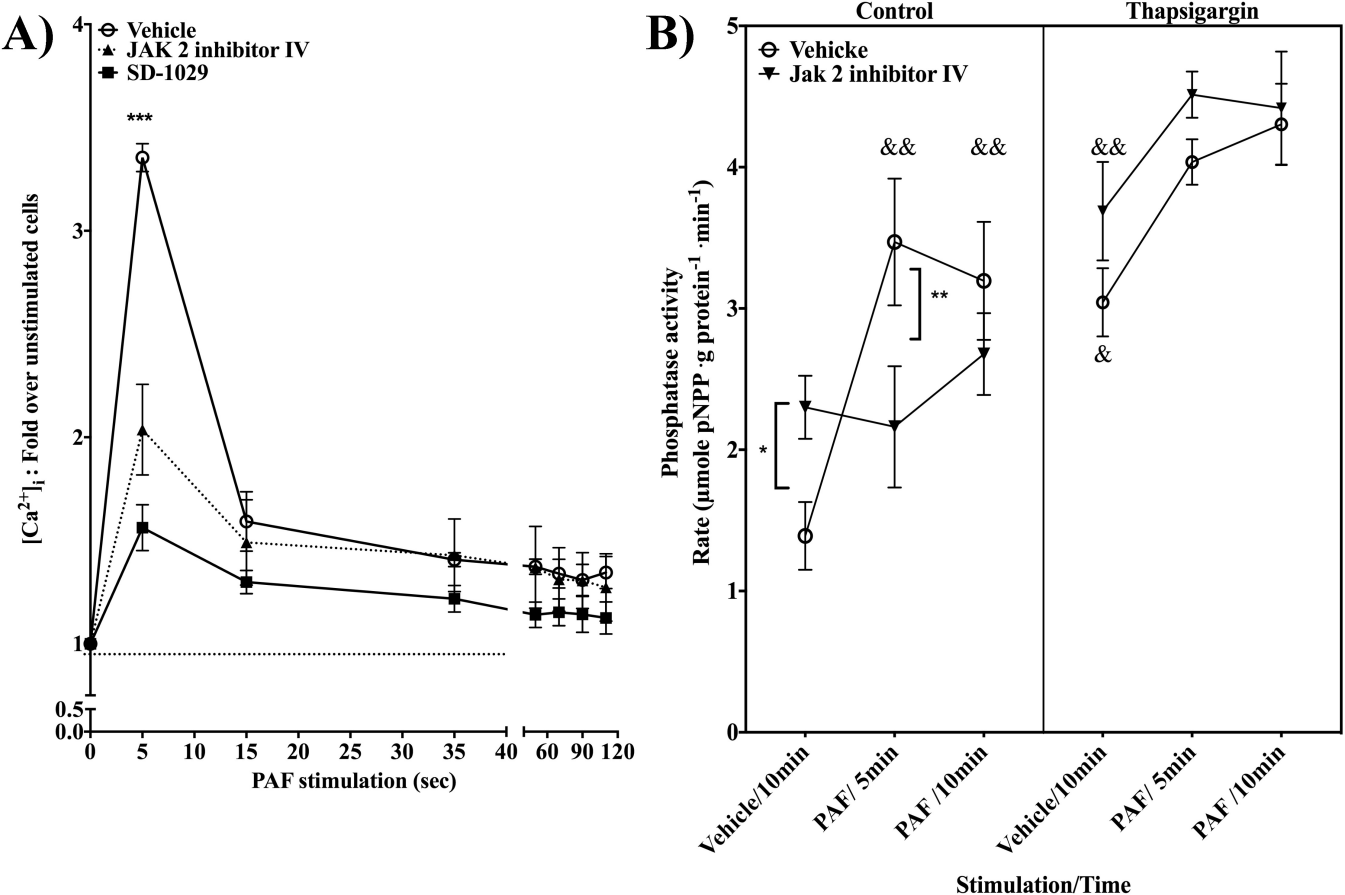
Jak2 regulates PTP1B in a calcium-dependent manner. **A)** HEK-PAFR were starved for 5h, incubated 30min with 5μM Fluo3 and pre-treated with vehicle, Jak2 inhibitor IV or SD-1029 for 20min in HBSS 0.2%BSA prior to stimulation with 100nM PAF in presence of 2mM CaCl_2_, for indicated times. Minimal and maximal fluorescence was determined by the addition of EGTA (100mM) or Triton X-100 (0,5% final), respectively. Data presented are mean±S.E.M of fold increase of [Ca^2+^]_i_ over unstimulated cells obtained from 3–4 independent experiments. Significance was established with paired two-way anova with Sidak post-test: *p<0.05, &p< 0.05 over unstimulated cells with the same pre-treatment. **B**) Phosphatase activity was measured via the hydrolysis rate of pNPP by PTP1B. HEK-PAFR were treated with Jak2 inhibitor IV or vehicle. These cells were then stimulated with vehicle, 200nM thapsigargin or 100nM PAF or co-stimulated with PAF and thapsigargin or PAF and vehicle. PTP1B was immunoprecipitated and incubated with pNPP. Data presented are mean ±S.E.M of hydrolysis rate from 4 independent experiments. Significance was established with paired two-way anova with Sidak post-test: ** p<0.01 and & p< 0.05 && p<0.01, over unstimulated cells with the same pre-treatment.

It is well known that calpain activation is regulated via the modulation of intracellular calcium concentrations ([Ca^2+^]_i_). Additionally, some studies show the involvement of Jak2 in [Ca^2+^]_i_ regulation [52]. Hence, we studied whether Jak2 could regulate [Ca^2+^]_i_ in PAF-stimulated cells and thus regulate PTP1B activity. HEK-PAFR were loaded with Fluo 3, pre-treated with Jak2 inhibitor IV or SD-1029 and stimulated with PAF. Changes in calcium levels were registered until 110sec post-stimulation. PAF induced a rapid increase in [Ca2+]i in control cells: 5s after stimulation, the [Ca^2+^]_i_ was 2432.9±315.1nM over the basal [Ca^2+^]_i_ (**Fig 11A**). On the other hand, in cells pre-treated with the Jak2 inhibitor IV or SD-1029, the [Ca^2+^]_i_ reached only 1157.3±165.3nM and 596.6±171.8nM, respectively, over basal levels, a significant decrease compared to control cells. For all conditions, the [Ca^2+^]_i_ rapidly decreased to similar levels around 35 sec. Pre-treatment with the Jak2 inhibitors did not significantly affect [Ca^2+^]_i_ in unstimulated cells and did not completely abrogate PAF-induced increase in [Ca^2+^]_i_. This latter point suggests that Jak2 is not essential for calcium response but rather that it is involved in the modulation of its intensity.

Next, we confirmed that modulation of PTP1B activity by Jak2 depended on calcium. PTP1B activity assays were performed using Jak2 inhibitor IV- or vehicle-pre-treated cells, which were then stimulated with 200nM thapsigargin or 100nM PAF or co-stimulated with PAF and thapsigargin. Co-stimulations were done to test whether the addition of thapsigargin could rescue PTP1B activity. **Fig 10B** shows that thaspsigargin alone increased pNPP hydrolysis rate by PTP1B, indicating that calcium mobilization alone is enough to influence PTP1B activation. This thapsigargin-mediated increase in pNPP hydrolysis rate was not affected by the Jak2 inhibitor IV. When cells were stimulated for 5 min with PAF, no difference in PTP1B activity was found between cells stimulated with PAF alone and those co-stimulated with thapsigargin. The hydrolysis rate, as described in **Fig 2**, was reduced in cells pre-treated with Jak2 inhibitor IV and stimulated 5min with PAF, but PTP1B activity was rescued when cells were co-stimulated with thapsigargin. These results suggest that Jak2 inhibitor IV-mediated decrease in calcium influx could cause the decrease seen in PAF-induced PTP1B activity in Jak2 inhibitor pre-treated cells. The PTP1B activity measured in Jak2 inhibitor IV-treated cells co-stimulated with PAF and thapsigargin was increased compared to co-stimulated cells without the Jak2 inhibitor IV, or compared to cells stimulated with PAF alone, suggesting that PAF could probably fine-tune PTP1B activity by other pathways than Jak2.

## Discussion

In atherosclerosis, PAF is found early in the onset of the plaque, produced by oxidized lipid-injured endothelial cells [5]. PAF allows the firm adhesion of monocytes to endothelial cells and their recruitment to the sub-endothelial site [7] where, under the action of cytokines, they differentiate into macrophages [53] or Mo-DCs. While the importance of macrophages in disease progression is well characterized, less it is known about Mo-DCs. A study with Langherans cells, a subset of Mo-DCs found in skin, has demonstrated that PAF stimulates IL-6 production [13], as had been shown in smooth muscle cells [17] and endothelial cells [54].

We have long been interested in PAFR-mediated signaling and our data, presented here, suggest that, in cells stimulated with PAF, PTP1B can be involved in negative feedback leading to control IL-6 mRNA expression in human iMo-DCs, suggesting a role for this PTP in modulating the inflammatory response. Results obtained with PTP1B immunoprecipitated from iMo-DCs indicate that this PTP is activated by PAF in a time-dependent manner, suggesting a possible involvement in negative feedback as PTP1B activation leads to the downregulation of PAF-stimulated IL-6 mRNA levels.

Even though we can not totally exclude that other PTPs could be co-immunoprecipitated with PTP1B, the results obtained with the in-gel PTPs assay, a much more sensitive method, indicated that PTP1B seems to be the only PTP immunoprecipitated in this experimental context, allowing us to conclude that the PTP activity seen in colorimetric PTP assays is due to PTP1B and that the contribution of other PTPs is negligible. In HEK-PAFR, as in iMo-DCs, PTP1B is involved in negative regulation of IL-6, since the pre-treatment of cells with the PTP1B inhibitor leads to an increase in PAF-induced IL-6 promoter activation. In addition, overexpression of PTP1B leads to a decrease in PAF-induced IL-6 promoter activity. Results obtained with the selective inhibitor also suggest that the regulatory effect of PTP1B depends on its PTP activity. Furthermore, our data also suggest that this modulation is partly dependent on Jak2 kinase activity since Jak2 inhibition, by selective inhibitors or transfection of a kinase-dead mutant, partly reverses the decrease of PAF-induced IL-6 promoter activity, caused by PTPIB. This rescue depends on PTP capacity of PTP1B since KD Jak2 cannot reverse inhibition of PAF-induced IL-6 promoter activation in cells transfected with the mutant D181A PTP1B. The fact that overexpression of WT and D181A PTP1B both lead to a decrease in PAF-induced IL-6 promoter activity could be surprising, but is coherent with other observations reported in the literature [35]. It is hypothesized that the prolonged binding of the PTP1B trapping mutant to its substrates hides phosphorylated docking sites or restrains interaction between other enzymes and the substrate, thus mimicking dephosphorylation [35].

In order to determine whether PTP1B modulated Jak2 activity, we first used confocal microscopy to determine whether Jak2 and PTP1B could be found in the same signaling complex. Then, we tracked PAF-induced Jak2 activity with antibodies against phospho-Tyr1007/1008 of Jak2, residues found in the activation loop. Microscopy done with Venus-tagged D181A PTP1B transfected-cells showed a rapid and transient interaction between Jak2 and the mutant PTP1B. Given that the interaction was transient with a mutant expected to "trap" Jak2 if PTP1B interacted with Jak2 via its phosphatase domain, we hypothesized that Jak2 may be found in the same complex but may not be the direct substrate of PTP1B in PAF-stimulated cells. Surprisingly, that although Jak2 has been identified as a PTP-1B substrate under many stimulatory conditions, stimulation with a GPCR, the PAFR, would place the two proteins in a complex where they may not interact directly, or Jak2 might not be directly in line with the phosphatase domain of PTP-1B.

We then pursued our studies with Western blots, which showed that PAF induced Jak2 phosphorylation in HEK-PAFR cells, as described in other cell types [31], but overexpression of WT or D181A mutant PTP1B did not alter the level of phospho-Tyr1007/1008 Jak2. To ensure that the absence of modulation of pJak2 levels in WT PTP1B-transfected cells was not due to a low transfection efficiency, we first monitored the transfection levels with GFP-PTP1B. As shown in **S6A Fig**, cells expressed GFP-WT PTP1B at significant levels 24h post-transfection. Compilation of 4 experiments done in sextuplicate showed that 52.8±2.37% of the cells expressed GFP-PTP1B. We also examined, in parallel with pJak2, the phosphorylation level of Src on Tyr527, also a known substrate of PTP1B [50]. HEK-PAFR were transfected with Flag-WT PTP1B or control vector and stimulated with 100nM PAF. As shown, in **S6B, S6C & S6D Fig**, PAF induced an increase in pTyr 527 levels at early stimulation times in control cells and, consistently with data from literature, this increase was significantly lower in WT-PT1B-transfected cells, suggesting that WT PTP1B transfection levels were sufficient to modulate PAF-induced signaling pathways. However, in those WT PTP1B-transfected cells, no modulation pJak2 was observed(**S6B Fig**). Thus, Western blots, microscopy and luciferase assays all lead us to conclude that PTP1B does not seems to modulate Jak2 activation. However, our results also show that PTP1B downregulation of PAF-induced hIL-6 promoter activity does seem to depend on Jak2 activity.

A recent study from Majumber et al. established a link between Jak2 activity and calpain-dependent vimentin cleavage [48]. Calpain, a calcium-dependent protease, is also known to regulate PTP1B cleavage and activation [49–50, 55–56]. Coincidentally, Jak2 has been shown to be involved in calcium regulation by diverse stimuli [52, 57], including CCL2-induced CXCR2 activation [58]. Altogether, these reports led us to hypothesize that Jak2 could modulate PAF-induced PTP1B activity in a calpain-dependent manner. To test this hypothesis, first, we demonstrated the involvement of Jak2 in PAF-induced PTP1B activation. PTP1B was immunoprecipitated from cells treated with 2 different Jak2 inhibitors and stimulated with PAF. Kinetic studies showed that, in control cells, PAF induced a rapid increase of PTP1B activity, visible as early as 2 min post-stimulation and sustained until 10 min. In the presence of both inhibitors, PAF failed to induce PTP1B activation and the hydrolysis rate stayed significantly lower than in control cells.

To further elucidate the mechanism by which Jak2 activates PTP1B, we investigated the calpain-mediated pathway. First, we ensured that upon PAF stimulation, PTP1B was cleaved and that this cleavage depended on Jak2. Results obtained with the two antibodies which recognize two different epitopes of PTP1B, suggested that PAF induced cleavage of PTP1B in a Jak2-dependent manner. Presence of a smaller PTP1B isoform was detected at early times of PAF stimulation. This isoform appeared only in control cells and not in Jak2 inhibitor-pre-treated cells. Furthermore, results obtained from in-gel PTP assays, with PTP1B immunoprecipitated from Mo-DCs and HEK-PAFR suggest that these isoforms may participate in the modulation of PAF-induced signaling pathways since their activity was higher in PAF-stimulated cells compared to control cells and this, in a Jak2-dependent manner.

The PAF-induced decrease in the 50kDa PTP1B band seems to be calpain-mediated, considering that this decrease is abrogated by pre-treatment with the calpain inhibitor, calpeptin. Next, we determined whether this cleavage had an activating or inhibiting effect on PTP1B activity, given that PTP1B cleavage may have contrasting effects, depending on the stimulation context [49–50, 55–56]. PTP assays with PTP1B immunoprecipitated from calpeptin-pre-treated cells suggest that calpain is involved in PAF-induced PTP1B activation. This activation of PTP1B by calpain is consistent with results obtained by others groups [50, 59]. However, others reported that calpain could also decrease PTP1B activity. In these reports, the decrease was due to an in vivo degradation of PTP1B [55] or degradation, following oxidation, which revealed a new cleavage site [49]. At the short times of stimulation used here, the activating effect of calpain-mediated cleavage of PTP1B was predominant, since we did not observe any degradation, given that the 42kDa fragment remained stable for all the tested times. Frangioni et al. also reported that the 42kDa fragment of PTP1B is stable for at least 30 min [56].

Supporting the importance of calpain in PTP1B activation, calpeptin partially rescued PAF-induced IL-6 promoter activation in PTP1B-transfected HEK-PAFR, as did Jak2 inhibition. This small, but significant increase in IL-6 promoter activation caused by calpeptin is observed in PTP1B-transfected HEK-PAFR whereas a decrease is found in control cells. This significant decrease in IL-6 promoter activity caused by calpain inhibition is consistent with a study performed in PBMC obtained from heathy or multiple-sclerosis donors that demonstrated a decrease in IL-6 mRNA in PBMC pre-treated with a calpain inhibitor and stimulated with anti-CD3/CD28 [60], suggesting that calpain could be involved in IL-6 production induced by different stimuli. Little is known about the involvement of calpain in PAF signaling. It has been reported that PAF could induce calpain 2 externalization in platelets, allowing calpain 2 to cleave its substrates and promote platelet aggregation [61]. Among substrates described in other systems, IκBα could be interesting as a calpain target in PAF-induced IL-6 promoter activation, given that it depends on NFκB activation and that calpain-mediated IκBα cleavage [62] could lead to the activation of this pathway.

Even though the intensity of PAF-induced calpain activation differed between experiments, our results suggest that PAF did transiently increase calpain activity, given that an increase was seen in all the experiments preformed. Our data do not allow us to determine the isoform of calpain activated. The rapid decrease in calpain activity showed a tight regulation of PAF-stimulated protease activity. The same activation pattern was achieved in iMo-DCs, suggesting that this mechanism of calpain regulation may not be cell type dependent. The exact mechanism for this transient activation in this model is not known but one could hypothesize that inhibition of calpain could be due to binding of calpastatin, an ubiquitously expressed unstructured peptide, to the Ca^2+^-calpain complex [62].

Jak2 inhibition, by two different inhibitors, abrogated PAF-induced calpain activation at early time points, suggesting that Jak2 kinase activation is a key step in the signaling pathway leading to calpain activation. A second, weaker, increase in calpain activity is observed at 30 min post-stimulation in both control and Jak2 inhibitor pre-treated cells. This could indicate that, at longer times, a Jak2-independent second wave of calpain activation would take place. It has been shown that Erk, also activated by PAF [63–65] could phosphorylate calpain 2 on serine 50, promoting is recruitment to the membrane and its activation by lower cytosolic calcium concentrations [66].

As calpain is known to be activated by calcium-dependent mechanisms and given that other groups have linked Jak2 to calcium influx [48, 52], we further investigated the signaling pathway by which Jak2 activates calpain by focusing on the involvement of Jak2 in PAF-induced calcium response. In HEK-PAFR, PAF stimulation increased intracellular calcium in a Jak2-dependent manner. These results are consistent with other studies. For example, it has been shown that leptin modulates calcium current in certain type of neurons by a L-type calcium channel-dependent mechanism where Jak2 could be involved [52]. It has also been demonstrated that erythropoietin increases intracellular calcium by a Jak2-dependent influx of extracellular calcium [67]. In CXCR4-induced calcium response, Jak2 is important for coupling Gα_i_ to CXCR4, allowing subsequent PLCβ activation [68]. Thus, although the exact mechanisms by which Jak2 modulates intracellular calcium levels remain unknown and further investigation will be need to elucidate it, one could hypothesize that Jak2 could phosphorylate certain calcium channels; in the same manner that Src phosphorylates certain L-type voltage-dependent calcium channels (Cav1.2b) in smooth muscle cells, thus enhancing calcium influx [69].

Furthermore, results obtained in this study establish a link between the impaired PAF-induced calcium response caused by Jak2 inhibition and the decrease in PTP1B activity. Effectively, in Jak2 inhibitor pre-treated cells, the co-stimulation with PAF and 200nM thapsigargin rescues PTP1B activity and this, without affecting the PTP1B activity in co-stimulated control cells. These results imply that PAF-induced intracellular calcium increase in control cells is sufficient and reaches the maximal [Ca^2+^]_i_ necessary for optimal PTP1B activation. Data from control cells also indicate that a rise in [Ca^2+^]_i_ alone, is sufficient to increase PTP1B activity, since thapsigargin alone is able to increase PTP1B activity and this, to similar levels as are observed with PAF stimulation. Different hypotheses could explain how co-stimulation with PAF and thapsigargin restores the PTP1B activity in Jak2 inhibitor pre-treated cells. First, it has been reported that PAF and thapsigargin sequentially added to peritoneal macrophages increased PAF-induced calcium store depletion, suggesting that the PAF-induced emptying of calcium stores is incomplete [70]. It is possible to hypothesize that in presence of activated Jak2, PAF-induced calcium depletion is sufficient to induce the opening of enough calcium channels to allow a calcium influx strong enough to activate PTP1B at the levels measured, given that in our hands, Jak2 seems to facilitate this influx. On the other hand, when Jak2 is inhibited, a more complete calcium store depletion is needed in order to generate a signal strong enough to allow opening of more channels, as Jak2 can not facilitate their opening. Thapsigargin could allow this depletion. Given that, in HEK-PAFR, PAF-induced calcium response is similar to the one observed in peritoneal macrophages (with a strong elevation followed by a rapid decrease and a weak, if not absent, plateau [70], contrasting to what is observed in other cell types (for example in microglial cells, where the increase is followed by an elevated and sustained plateau under control of store-operated channels) [19]. A parallel between PAF-induced calcium response in HEK and macrophages could be suggested, but still keeping in mind that they are very different cell types. As a second hypothesis, given that thapsigargin is known to be able to prolong calcium influx by, among other ways, activating the Ca^2+^/calmodulin pathway, maintaining Ca^2+^-dependent calcium permeable channels opened for longer time [71], co-stimulation with thapsigargin could promote extended influx. This prolongation could be enough to compensate for the decrease in calcium influx caused by Jak2 inhibition. Further investigation is needed to elucidate which type of calcium response is modulated by Jak2. Study of PAF-induced calcium response in murine 2-cell embryos suggests that PAF-induced calcium influx is predominantly via voltage-gated L-type calcium channels, as demonstrated by pharmacological inhibitors [72]. Also, in smooth muscle cells, pharmacological inhibition of voltage-gated L-type calcium channels decreased PAF-induced IL-6 production [17], suggesting that this type of calcium channel could also be a target of Jak2-regulated calcium influx. Store-operated channels have also been involved in PAF-induced calcium response [19] and could also be another pathway to investigate.

In conclusion, we report here, for the first time, that PAF activates PTP1B via a Jak2- and Ca2+-dependent pathway and that this activation results in the fine regulation of PAF-induced IL-6 mRNA production. Our results indicate that PAF-stimulated Jak2, via the modulation of PAF-induced calcium responses, in a calpain-dependent manner, regulates PAF-induced PTP1B activation, setting up a negative feedback loop for IL-6 production.

## Supporting information

S1 Fig PAF induces PTP1B activity. **A)** Representative autoradiography with lysates of HEK-PAFR stimulated with PAF. Cells were starved overnight in serum-free medium with 0.2%BSA and stimulated with 100nM PAF for indicated times. Cells were lysed with in-gel PTP lysis buffer and 15μg of whole cell lysate was loaded onto a 10% acrylamide SDS-PAGE gel, copolymerized with labeled poly(Glu-:Tyr). **B)** Graph summarizing the normalized intensity of bands according to the molecular weight and stimulation time. Data presented are mean ±S.E.M of normalized intensity (as described in Materials & Methods) for 3–4 independent experiments.
(TIF)

S2 Fig Jak2 is involved in PAF-induced PTP1B activity. We studied PAF-stimulated phosphatase activity in the presence of a Jak2 inhibitor. Graph represents the hydrolysis rate of pNPP by PTP1B immunoprecipitated from HEK-PAFR stimulated with 100nM PAF for indicated times, after pre-treatment with 0.2μM SD-1029. PTP1B was immuoprecipitated from HEK-PAFR, which had been starved overnight in DMEM 0.2% BSA, pre-treated and stimulated as indicated before being incubated with pNPP. Data presented are mean ±S.E.M of pNPP hydrolysis rate for at least 3 independent experiments. Significance was established with paired two-way anova with Sidak post-test: p<0.05, ** p<0.01 and &:p< 0.05 over unstimulated cells with the same pre-treatment.
(TIF)

S3 Fig Jak2 regulates PTP1B cleavage. HEK-PAFR were starved overnight and pre-treated with vehicle or 0.2μM SD-1029 for 20min prior to stimulation with 100nM PAF for indicated times. Whole cell lysates were loaded onto SDS-PAGE, transferred to nitrocellulose membranes and blotted overnight with indicated antibodies, mouse anti-PTP1B, epitope 301–435 or goat anti-PTP1B, epitope 2–321 and the decrease of PTP1B expression was determined. **A)** Representative Western blots of PTP1B levels are shown for mouse anti-PTP1B, epitope 301–435 and goat anti-PTP1B, epitope 2–321. Blots were scanned and the variations in PTP1B levels are represented as ratio of PTP1B expression in stimulated cells over control, untreated and unstimulated cells. Data presented are mean±S.E.M of 3 independent experiments. Significance was established with paired two-way anova with Sidak post-test: *p<0.05, &:p< 0.05 over unstimulated cells with the same pre-treatment.
(TIF)

S4 Fig PAF induces PTP1B cleavage in a calpain-dependent manner. **A)** Representative western blot of PTP1B levels. HEK-PAFR were starved overnight, then pre-treated with 70nM calpeptin or vehicle for 20min prior to stimulation with 100nM PAF for indicated times. Whole cell lysates were loaded onto SDS-PAGE, transferred onto nitrocellulose membrane and blotted overnight with mouse anti-PTP1B, eptiope 301–435. **B)** Data are presented as relative intensity of PTP1B expression over vinculin and reported as fold change at indicated times. Data presented are mean±S.E.M of 5–7 independent experiments. Significance was established with paired two-way anova with Sidak post-test: *p<0.05, &:p< 0.05 over unstimulated cells with the same pre-treatment.
(TIF)

S5 Fig Jak2 is involved PAF-induced calpain activity. HEK-PAFR were starved overnight and pre-treated with vehicle or 0.2μM SD-1029 for 20min prior to stimulation with 100nM PAF for indicated times and lysis with Fluorometric Activity Assay Kit lysis buffer. Assays were performed according to manufacturer's instruction. Variations in calpain activity levels are presented as ratio of fluorescence of stimulated cells over untreated, unstimulated cells. Data presented are mean±S.E.M of 3 independent experiments. Significance was established with paired two-way anova with Sidak post-test: *p<0.05, &:p< 0.05 over unstimulated cells with the same pre-treatment.
(TIF)

S6 Fig PTP1B modulates PAF-induced pSrc without affecting pJak2. **A)** A representative experiment showing GFP2-WT PTP1B expression levels, 24h post-transfection. HEK-PAFR were transfected with 0,67μg pGFP2-PTP1B plasmid per wells of a 6 well plate, same as Flag-WT PTP1B. 24h post-transfection, cells were collected and PTP1B expression was determined by flow cytometry using untransfected cells as control for basal fluorescence. **B, C & D)** HEK-PAFR were transfected with Flag-tagged WT PTP1B or control vector for 8 h before overnight starvation in DMEM, 0.2% BSA. Cells were stimulated for indicated times with PAF (100nM) or vehicle. Whole cell lysates were loaded onto SDS-PAGE, transferred onto nitrocellulose membrane and blotted overnight with rabbit anti-pJak2 Tyr 1007/1008, rabbit anti-pTr 527 Src, then stripped and bloted with rabbit anti-Jak2, anti-Src and anti-Flag. **B**) Representative blots **C &D)** Blots were scanned and the variation in **C)** Jak2 phosphorylation (pJak2 Tyr 1007/1008) or **D)** pSrc (pTyr 527 Src) are represented as ratio of phosphorylation in stimulated cells over control unstimulated cells. Data presented are mean±S.E.M of 3 independent experiments. Significance was established with paired two-way anova with Sidak post-test. *: p<0.05.
(TIF)
