# Supplementary figures and images for: Regulation of platelet-activating factor-mediated protein tyrosine phosphatase 1B activation by a Janus kinase 2/calpain pathway
Source: PLoS One. 2017 Jul 7;12(7):e0180336. doi: 10.1371/journal.pone.0180336 (PMC5501562; doi:10.1371/journal.pone.0180336)

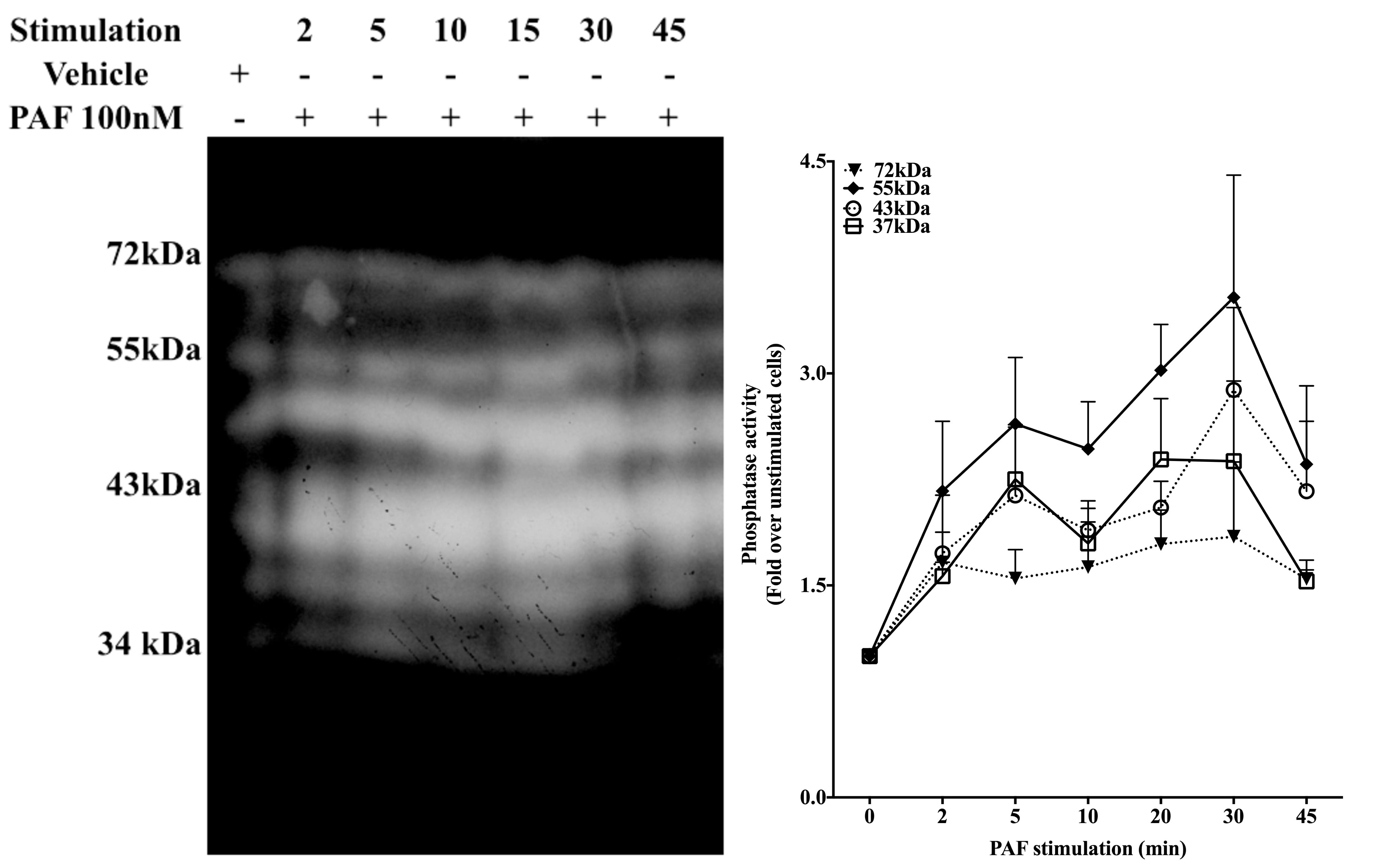

Supplement: S1 Fig — A) Representative autoradiography with lysates of HEK-PAFR stimulated with PAF. Cells were starved overnight in serum-free medium with 0.2%BSA and stimulated with 100nM PAF for indicated times. Cells were lysed with in-gel PTP lysis buffer and 15μg of whole cell lysate was loaded onto a 10% acrylamide SDS-PAGE gel, copolymerized with labeled poly(Glu-:Tyr). B) Graph summarizing the normalized intensity of bands according to the molecular weight and stimulation time. Data presented are mean ±S.E.M of normalized intensity (as described in Materials & Methods) for 3–4 independent experiments. (TIF) [file pone.0180336.s001.tif]

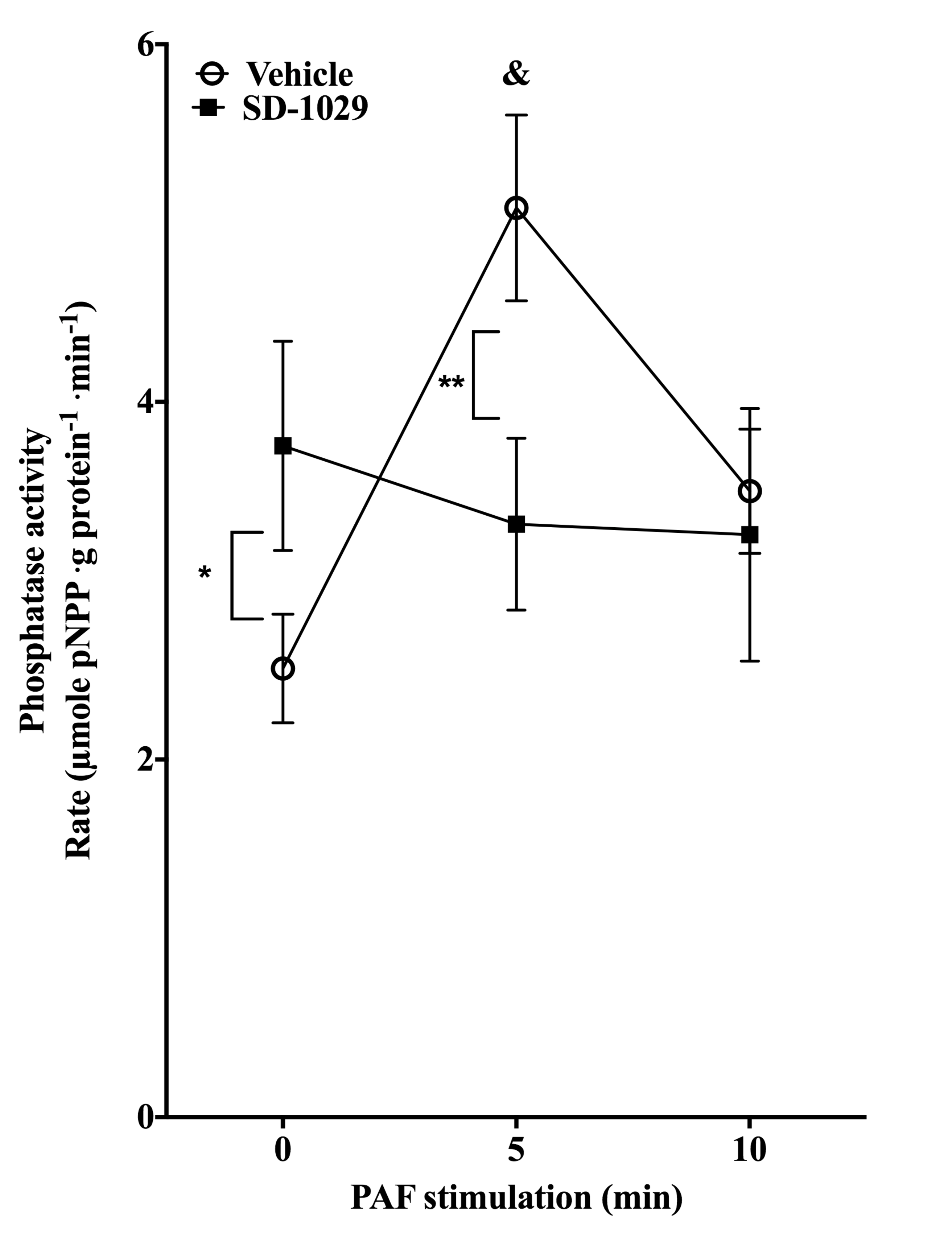

Supplement: S2 Fig — We studied PAF-stimulated phosphatase activity in the presence of a Jak2 inhibitor. Graph represents the hydrolysis rate of pNPP by PTP1B immunoprecipitated from HEK-PAFR stimulated with 100nM PAF for indicated times, after pre-treatment with 0.2μM SD-1029. PTP1B was immuoprecipitated from HEK-PAFR, which had been starved overnight in DMEM 0.2% BSA, pre-treated and stimulated as indicated before being incubated with pNPP. Data presented are mean ±S.E.M of pNPP hydrolysis rate for at least 3 independent experiments. Significance was established with paired two-way anova with Sidak post-test: p<0.05, ** p<0.01 and &:p< 0.05 over unstimulated cells with the same pre-treatment. (TIF) [file pone.0180336.s002.tif]

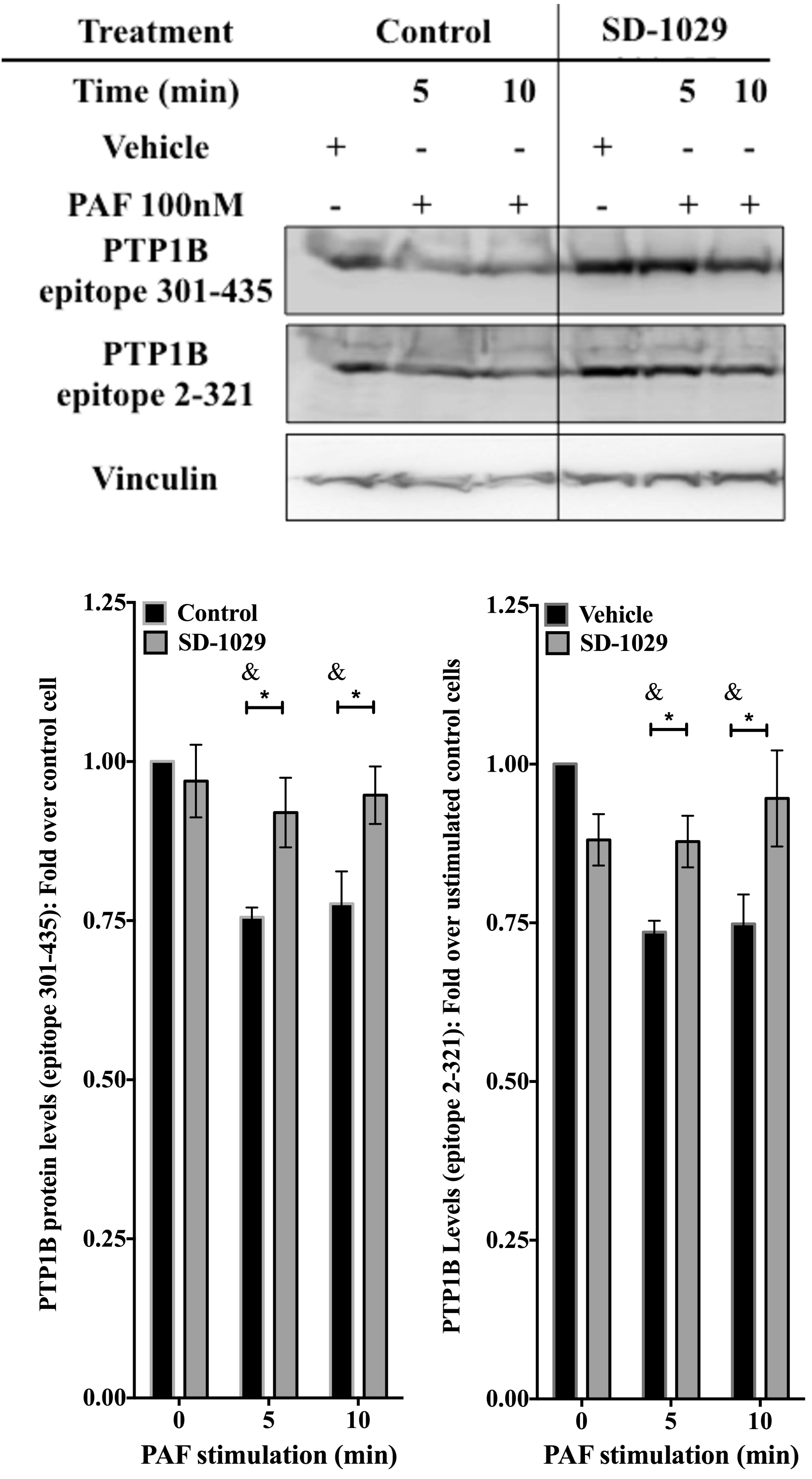

Supplement: S3 Fig — HEK-PAFR were starved overnight and pre-treated with vehicle or 0.2μM SD-1029 for 20min prior to stimulation with 100nM PAF for indicated times. Whole cell lysates were loaded onto SDS-PAGE, transferred to nitrocellulose membranes and blotted overnight with indicated antibodies, mouse anti-PTP1B, epitope 301–435 or goat anti-PTP1B, epitope 2–321 and the decrease of PTP1B expression was determined. A) Representative Western blots of PTP1B levels are shown for mouse anti-PTP1B, epitope 301–435 and goat anti-PTP1B, epitope 2–321. Blots were scanned and the variations in PTP1B levels are represented as ratio of PTP1B expression in stimulated cells over control, untreated and unstimulated cells. Data presented are mean±S.E.M of 3 independent experiments. Significance was established with paired two-way anova with Sidak post-test: *p<0.05, &:p< 0.05 over unstimulated cells with the same pre-treatment. (TIF) [file pone.0180336.s003.tif]

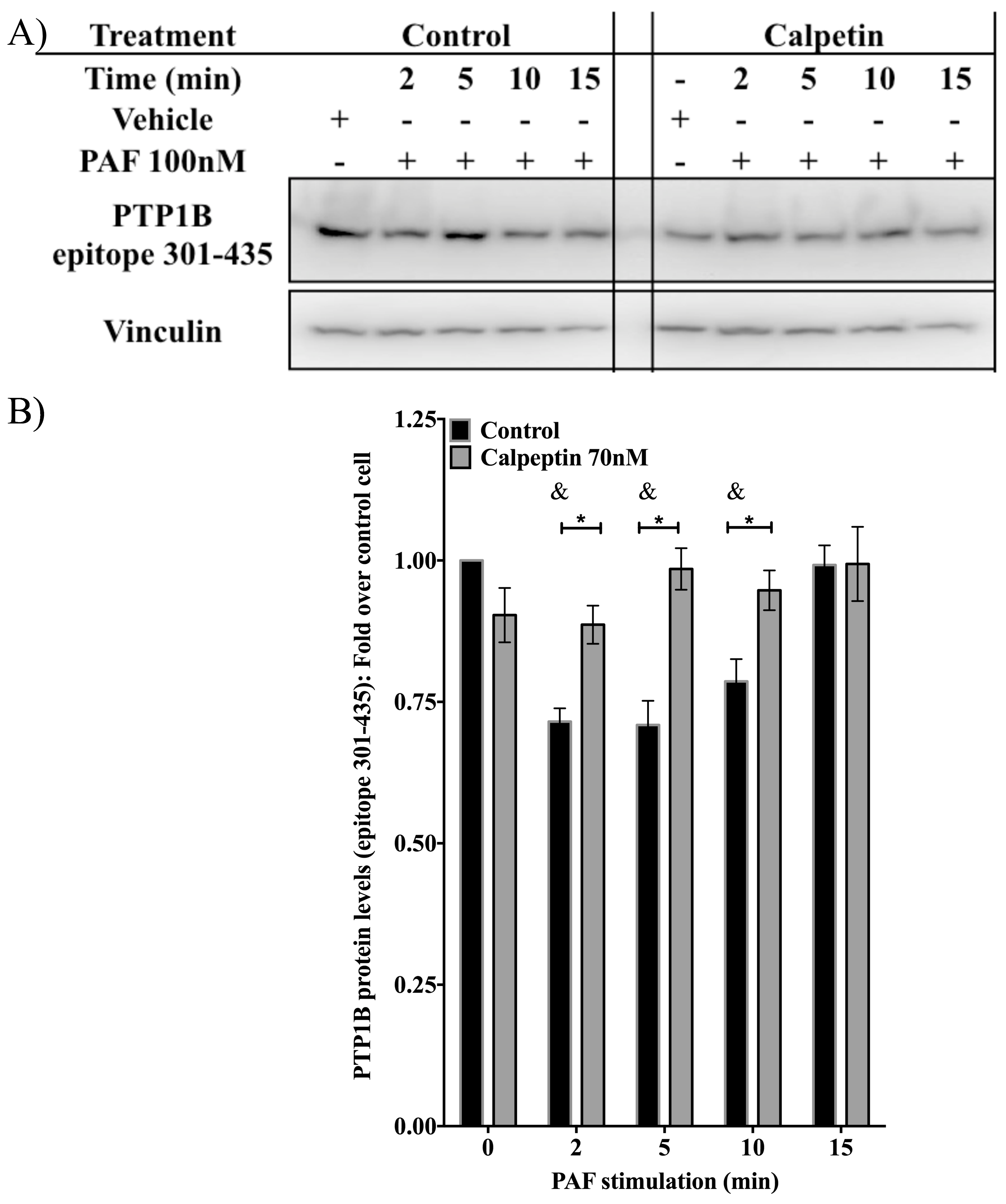

Supplement: S4 Fig — A) Representative western blot of PTP1B levels. HEK-PAFR were starved overnight, then pre-treated with 70nM calpeptin or vehicle for 20min prior to stimulation with 100nM PAF for indicated times. Whole cell lysates were loaded onto SDS-PAGE, transferred onto nitrocellulose membrane and blotted overnight with mouse anti-PTP1B, eptiope 301–435. B) Data are presented as relative intensity of PTP1B expression over vinculin and reported as fold change at indicated times. Data presented are mean±S.E.M of 5–7 independent experiments. Significance was established with paired two-way anova with Sidak post-test: *p<0.05, &:p< 0.05 over unstimulated cells with the same pre-treatment. (TIF) [file pone.0180336.s004.tif]

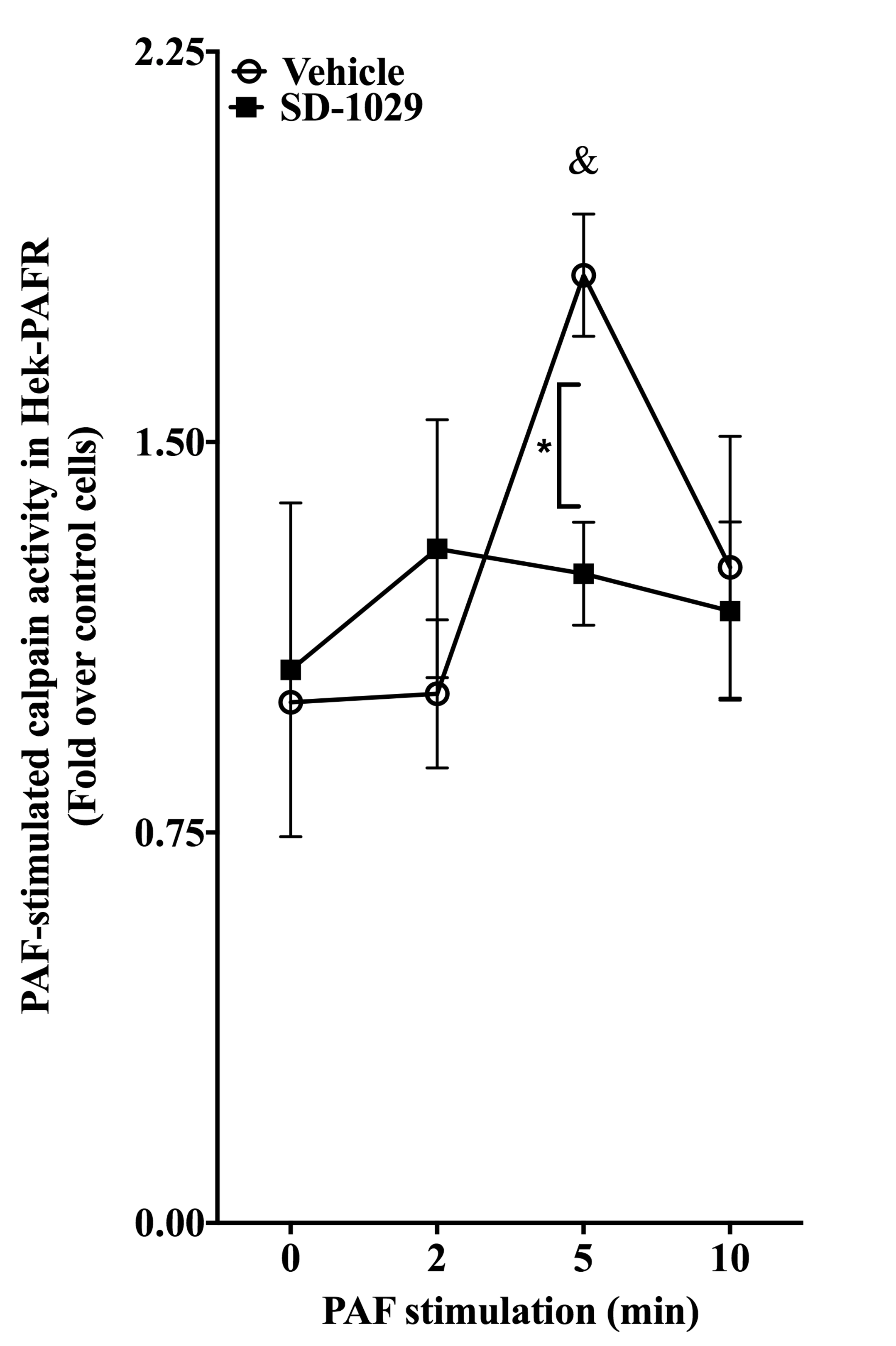

Supplement: S5 Fig — HEK-PAFR were starved overnight and pre-treated with vehicle or 0.2μM SD-1029 for 20min prior to stimulation with 100nM PAF for indicated times and lysis with Fluorometric Activity Assay Kit lysis buffer. Assays were performed according to manufacturer's instruction. Variations in calpain activity levels are presented as ratio of fluorescence of stimulated cells over untreated, unstimulated cells. Data presented are mean±S.E.M of 3 independent experiments. Significance was established with paired two-way anova with Sidak post-test: *p<0.05, &:p< 0.05 over unstimulated cells with the same pre-treatment. (TIF) [file pone.0180336.s005.tif]

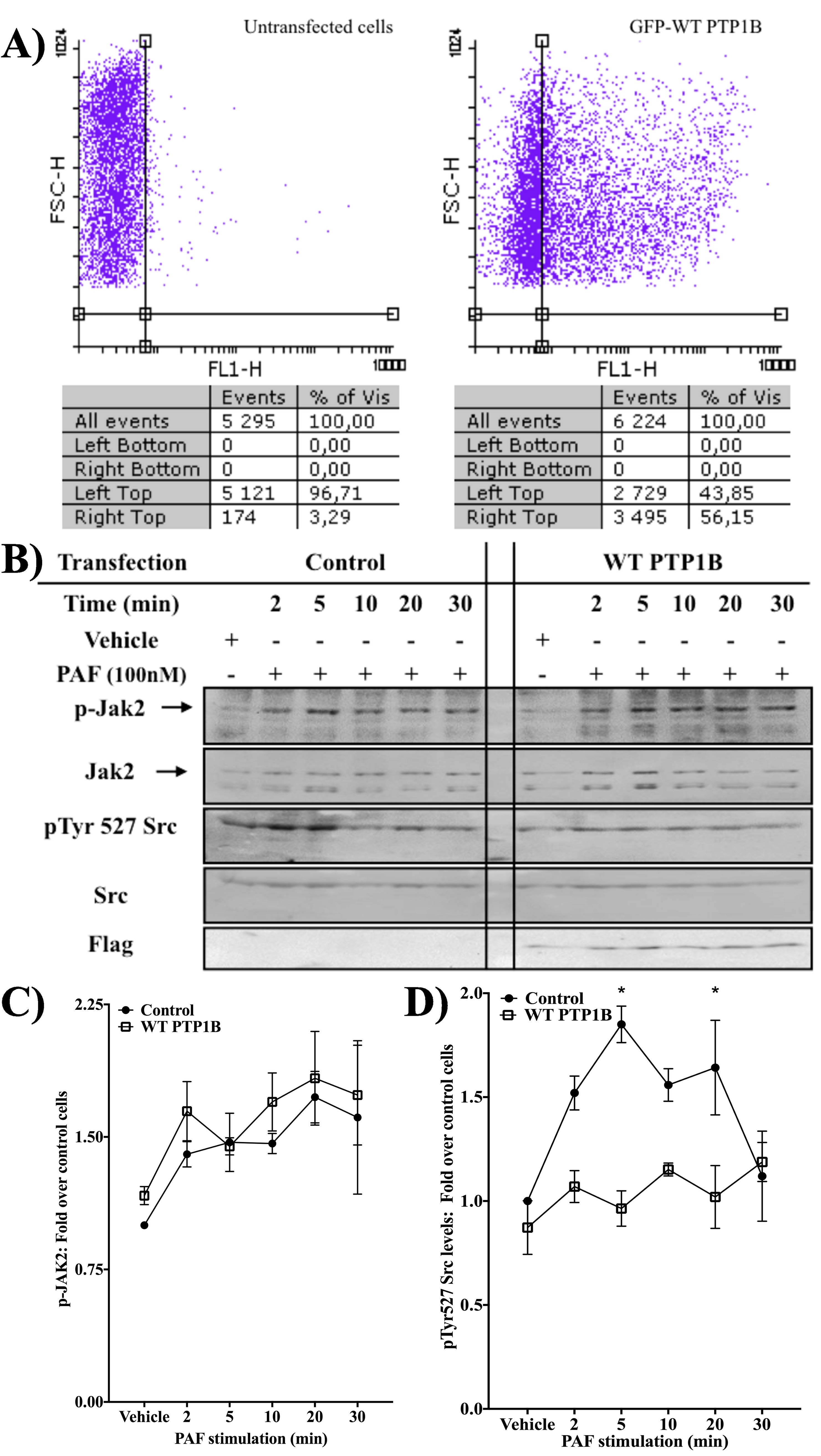

Supplement: S6 Fig — A) A representative experiment showing GFP2-WT PTP1B expression levels, 24h post-transfection. HEK-PAFR were transfected with 0,67μg pGFP2-PTP1B plasmid per wells of a 6 well plate, same as Flag-WT PTP1B. 24h post-transfection, cells were collected and PTP1B expression was determined by flow cytometry using untransfected cells as control for basal fluorescence. B, C & D) HEK-PAFR were transfected with Flag-tagged WT PTP1B or control vector for 8 h before overnight starvation in DMEM, 0.2% BSA. Cells were stimulated for indicated times with PAF (100nM) or vehicle. Whole cell lysates were loaded onto SDS-PAGE, transferred onto nitrocellulose membrane and blotted overnight with rabbit anti-pJak2 Tyr 1007/1008, rabbit anti-pTr 527 Src, then stripped and bloted with rabbit anti-Jak2, anti-Src and anti-Flag. B) Representative blots C &D) Blots were scanned and the variation in C) Jak2 phosphorylation (pJak2 Tyr 1007/1008) or D) pSrc (pTyr 527 Src) are represented as ratio of phosphorylation in stimulated cells over control unstimulated cells. Data presented are mean±S.E.M of 3 independent experiments. Significance was established with paired two-way anova with Sidak post-test. *: p<0.05. (TIF) [file pone.0180336.s006.tif]
